# The effects of hypothetical behavioral interventions on the 13-year incidence of overweight/obesity in children and adolescents

**DOI:** 10.1186/s12966-023-01501-6

**Published:** 2023-08-24

**Authors:** C. Börnhorst, I. Pigeot, S. De Henauw, A. Formisano, L. Lissner, D. Molnár, L. A. Moreno, M. Tornaritis, T. Veidebaum, T. Vrijkotte, V. Didelez, M. Wolters, on behalf of the GrowH! consortium

**Affiliations:** 1 Leibniz Institute for Prevention Research and Epidemiology - BIPS, Achterstr 30, 28359 Bremen, Germany; 2 Institute of Statistics, Faculty of Mathematics and Computer Science, University of Bremen, Bremen, Germany; 3 Department of Public Health and Primary Care, Ghent University, Ghent, Belgium; 4 Institute of Food Sciences, National Research Council, Avellino, Italy; 5 School of Public Health and Community Medicine, Institute of Medicine, Sahlgrenska Academy, University of Gothenburg, Gothenburg, Sweden; 6 Department of Pediatrics, Medical School, University of Pécs, Pécs, Hungary; 7 GENUD (Growth, Exercise, Nutrition and Development) Research Group, Faculty of Health Sciences, Universidad de Zaragoza, Instituto Agroalimentario de Aragón (IA2), Instituto de Investigación Sanitaria Aragón (IIS Aragón), Saragossa, Spain; 8 Centro de Investigación Biomédica en Red de Fisiopatología de La Obesidad Y Nutrición (CIBERObn), Madrid, Spain; 9 Research and Education Institute of Child Health, Strovolos, Cyprus; 10 National Institute for Health Development, Estonian Centre of Behavioral and Health Sciences, Tallinn, Estonia; 11 Department of Public and Occupational Health, Amsterdam University Medical Center (Amsterdam UMC), University of Amsterdam, Amsterdam, The Netherlands

**Keywords:** Causal inference, Childhood obesity, IDEFICS/I.Family cohort, Modifiable risk factor, Observational data, Parametric g-formula

## Abstract

**Background:**

In view of the high burden of childhood overweight/obesity (OW/OB), it is important to identify targets for interventions that may have the greatest effects on preventing OW/OB in early life. Using methods of causal inference, we studied the effects of sustained behavioral interventions on the long-term risk of developing OW/OB based on a large European cohort.

**Methods:**

Our sample comprised 10 877 children aged 2 to < 10 years at baseline who participated in the well-phenotyped IDEFICS/I.Family cohort. Children were followed from 2007/08 to 2020/21. Applying the parametric g-formula, the 13-year risk of developing OW/OB was estimated under various sustained hypothetical interventions on physical activity, screen time, dietary intake and sleep duration. Interventions imposing adherence to recommendations (e.g. maximum 2 h/day screen time) as well as interventions ‘shifting’ the behavior by a specified amount (e.g. decreasing screen time by 30 min/day) were compared to ‘no intervention’ (i.e. maintaining the usual or so-called natural behavior). Separately, the effectiveness of these interventions in vulnerable groups was assessed.

**Results:**

The 13-year risk of developing OW/OB was 30.7% under no intervention and 25.4% when multiple interventions were imposed jointly. Meeting screen time and moderate-to-vigorous physical activity (MVPA) recommendations were found to be most effective, reducing the incidence of OW/OB by -2.2 [-4.4;-0.7] and -2.1 [-3.7;-0.8] percentage points (risk difference [95% confidence interval]), respectively. Meeting sleep recommendations (-0.6 [-1.1;-0.3]) had a similar effect as increasing sleep duration by 30 min/day (-0.6 [-0.9;-0.3]). The most effective intervention in children of parents with low/medium educational level was being member in a sports club; for children of mothers with OW/OB, meeting screen time recommendations and membership in a sports club had the largest effects.

**Conclusions:**

While the effects of single behavioral interventions sustained over 13 years were rather small, a joint intervention on multiple behaviors resulted in a relative reduction of the 13-year OW/OB risk by between 10 to 26%. Individually, meeting MVPA and screen time recommendations were most effective. Nevertheless, even under the joint intervention the absolute OW/OB risk remained at a high level of 25.4% suggesting that further strategies to better prevent OW/OB are required.

**Supplementary Information:**

The online version contains supplementary material available at 10.1186/s12966-023-01501-6.

## Introduction

Various recommendations exist for health-related behaviors such as sleep duration, media time, physical activity (PA) or diet [1–7]. However, to date, little is known on long-term effects of sustained adherence to such recommendations on overweight/obesity (OW/OB). Randomized controlled trials (RCT) are typically not feasible over long time spans, for cost, time, practical or ethical reasons and adherence is difficult to enforce. This is particularly true for studies in children and adolescents [8]. Indeed, most intervention studies in young populations, e.g. on PA and diet, covered less than one year with only a few lasting more than two years, and even the latter only showed small effects on body mass index (BMI) [9–11]. Therefore, other approaches are required to assess the long-term effects of adherence to health-related recommendations on the weight status. The rationale of our study is to apply methods of causal inference to observational data to fill this gap. We use the so-called parametric g-formula [12] to answer the research question ‘What would happen to the incidence of OW/OB if all children continually adhered to behavioral recommendations of official bodies such as the Center for Disease Control over a period of 13 years?’.

Previous observational studies such as large cohort studies investigating the role of modifiable factors in the development of OW/OB yielded mixed results [13–15]. In addition, conventional analyses of observational data often ignore the problem of time-varying confounding when the target behavior/exposure needs to be sustained over a time-period. Standard regression adjustment is biased in the presence of time-varying confounding, i.e. if the confounders for future exposure are themselves affected by past exposure. The so-called g-methods solve this problem under certain structural assumptions [12]. The parametric g-formula, a generalization of standardization to time-varying settings, is particularly suitable for research questions about sustained, time-dependent or adaptive exposure effects. It can be used to estimate the effects of single or combined hypothetical intervention strategies while appropriately adjusting for time-varying confounding. To date, there are only few studies using methods of causal inference to estimate long-term effects of hypothetical interventions on childhood OW/OB from observational data [16–18].

Our study improves on previous work by explicitly defining hypothetical intervention strategies and assessing their effects under full adherence on the 13-year risk of developing OW/OB from infancy to young adulthood based on a large well-phenotyped European children cohort. Furthermore, we compare the effects of ‘no intervention’ (i.e. children maintain their usual or so-called natural behavior) not only to interventions imposing adherence to recommendations (e.g. maximum 2 h/day screen time) but also to interventions ‘shifting’ the behavior by a specified amount (e.g. decreasing screen time by 30 min/day). ‘Shift’ interventions may be attractive as they typically require smaller behavioral changes, and may hence be easier to achieve. Previous hypothetical intervention studies in children were based on smaller samples, covered rather short time periods and evaluated intervention effects mainly based on recommendations [16, 17]. In addition, we study the effectiveness of hypothetical interventions when targeting children from vulnerable groups, i.e. children of families with low or medium educational level and children of mothers with OW/OB. Low parental educational attainment [19, 20] and maternal OW/OB [21] are known risk factors for unhealthy behaviors and might predispose to a high vulnerability for OW/OB in children.

Our study aims to answer the following *research question*: (i) What are the single and joint effects on the 13-year incidence of OW/OB if all children continually adhered to behavioral interventions regarding screen time, membership in a sports club, using active forms of transport, moderate-to-vigorous physical activity (MVPA), sleep duration, consuming sugar-sweetened beverages (SSB) and meal time distractions. Secondary research questions are: (ii) Which of the considered interventions, individually, are the most effective ones; (iii) Do shift interventions have similar effects compared to interventions requiring adherence to recommendations; (vi) Which interventions are specifically promising in vulnerable groups? We expect our results to inform policy makers, health authorities, health professionals and educators, pediatricians, and family caregivers.

## Methods

### The IDEFICS/I.Family cohort

The IDEFICS/I.Family cohort is a multi-center population-based study aiming to investigate the causes of diet- and lifestyle-related diseases in children, adolescents and their families [22, 23]. The baseline survey wave (W0) was conducted in 2007/2008 in eight European countries (Belgium, Cyprus, Estonia, Germany, Hungary, Italy, Spain and Sweden) and included children aged 2 to 9 years. In total, 16 229 children fulfilling the inclusion criteria participated. The survey included interviews with parents concerning lifestyle habits of their children such as dietary intakes, physical activity and sedentary behaviors, as well as physical examinations and the collection of blood samples. All measurements and samples were taken using standardized procedures in all eight countries. Additional details on the IDEFICS/I.Family study can be obtained from Ahrens et al. [22, 23]. A follow-up examination (W1) was conducted in 2009/2010 with the same standardized assessments in 11 293 children participating already in W0. A second follow-up examination (W2) took place in 2013/2014, where 6242 of those children were included. In 2020/2021, a web-based follow-up (W3) was conducted with 5073 subjects participating. In W2 and W3, study subjects aged 12 years or older self-reported their lifestyle behaviors, well-being and family life.

Before children entered the study, parents provided written informed consent for their children. Additionally, all children aged 12 years and older gave written consent, while younger children gave oral assent in addition to parental consent for the examinations and sample collection. Ethical approval was obtained from the institutional review boards of all eight study centers. The IDEFICS/I.Family cohort is registered under ISRCTN62310987.

### Eligibility criteria

**Fig. 1:**
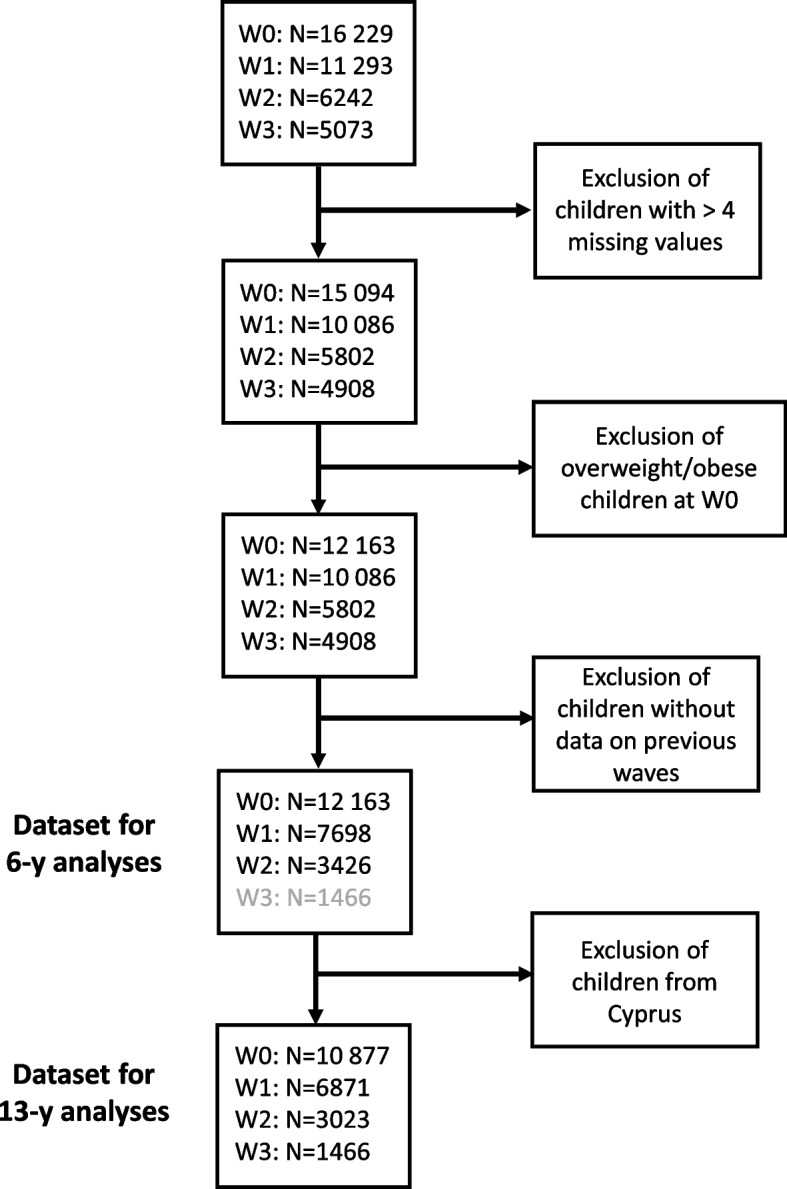
Flow chart depicting the selection process leading to the datasets used in the main analyses (13-y analyses) and the analyses restricted to W0 to W2 data (6-y analyses)

Our sample comprised all children who participated in the baseline survey and were not overweight or obese at baseline. Children who first entered the study in later waves were not included, nor data of children from waves with more than four missing values in exposures or covariates. As no data from children in Cyprus were available in W3, these were excluded from any analyses using data from W3. Figure 1 depicts the selection process leading to the final study sample of 10 877 children (6871 at W1, 3023 at W2 and 1466 at W3) in the main analyses covering a 13-year period, and 12 163 children in the analyses restricted to W0 to W2 covering a 6-year period. Additional analyses were performed in a subgroup of 2203 children for whom information on objectively measured moderate-to-vigorous physical activity (MVPA) from accelerometers was available.

### Outcome

Our primary outcome was the 13-year risk of developing OW/OB. The secondary outcome was the 6-year risk of developing OW/OB.

### Modifiable behaviors (exposures) targeted by hypothetical interventions

We focused on seven behavioral exposures: nocturnal sleep duration (hours/day), screen time (hours/day; including TV and PC time at W0 and W1; TV, PC and web time at W2 and W3), sugar-sweetened beverages (excluding sugar-sweetened milk and fruit juices), eating while doing something else (e.g. watching TV; dichotomized as ‘daily’ vs ‘non-daily’), membership in sports club (yes vs no), active transport to/from kindergarten/school/work (yes vs no) and in a subgroup accelerometer-measured MVPA (min/day).

### Hypothetical intervention strategies

For all continuous exposures, we defined two types of strategies: 1) an intervention according to recommendations as given by official bodies such as the Center for Disease Control, the American Psychological Association or the American Academy of Pediatrics (see Table 1), and 2) a shift intervention changing the natural exposure values by a specified amount to mimic perhaps more feasible interventions. For binary exposures, interventions were assumed to change the variable values to the favorable one (e.g. ‘all children are members in a sports club’). All interventions were sustained during the whole follow-up period, i.e. we were interested in the per-protocol effect under full adherence. The specific interventions are summarized in Table 1.

**Table 1:** Intervention variables and definition of interventions according to recommendations and shift interventions

Intervention variable	Intervention according to recommendation		Shift intervention
Sleep duration^a^	All children adhere to age-specific sleep recommendations at all waves:		Sleep duration is increased in all children that do not meet the lower recommended level by 0.5 h
	Age	Recommended sleep duration	
	3–5 years	10–13 h per 24 h (incl. naps)^f^	
	6–12 years	9–12 h per 24 hours^f^	
	13–18 years	8–10 h per 24 hours^f^	
	18–60 years	7 or more hours per night^g^	
	If child sleeps less than recommended, sleep duration of that child is set to the lower value of the recommendation		
Screen time^b^	All children < 6 years have a screen time of no more than 1 h/day All children ≥ 6 years have a screen time of no more than 2 h/day If child has higher screen time than recommended, screen time is set to upper the value of the recommendation		If children do not meet screen time recommendations, screen time is reduced by 0.5 h or, if recommendation is exceeded by less than 0.5 h, screen time is set to upper level of recommendation
Sugar-sweetened beverage consumption^c^	All children drink SSB no more than 4 times/week If child consumes SSB > 4 times/week, SSB is set to 4 times/week		If SSB > 4 times/week, SSB is reduced by 1 per day but not below 4 times/week
Eating while doing something else^d^	All children eat while doing something else on a non-daily basis, i.e. less than daily If child eats while doing something else ‘daily’, value is set to ‘non-daily’		
Membership in sports club^d^	All children are a member of a sports club		
Active transport^d^	All children and adolescents use an active form of transport to/from kindergarten/school/work		
MVPA^e^	All children meet PA recommendations of at least 60 min MVPA per day If MVPA < 60 min/day, then MVPA is set to 60 min/day		If children do not meet MVPA recommendations, MVPA is increased by 15 min/day or, if recommendation is underrun by less than 15 min/day, MVPA is set to lower level of recommendation (60 min/day)

^a^Recommendations according to Center for Disease Control 2017 [3]

^b^Recommendations according to American Psychological Association 2019 [4] and American Academy of Pediatrics [5]

^c^No clear recommendations exist but a recent review and meta-analysis pointed to an unfavorable effect of consuming more than 4 SSB/week [24]

^d^*Membership in sports club, active transport* and *eating while doing something else* were dichotomous variables and no clear recommendations exist. For these reasons, here interventions were defined as adhering to the assumed favorable level

^e^Recommendations simplified according to World Health Organization for children under 5 years 2019 [6] and for children aged ≥ 5 years of age and youth 2020 [7]

^f^Recommendations based on a consensus statement of the American Academy of Sleep Medicine [2]

^g^Recommendations based on a joint consensus statement of the American Academy of Sleep Medicine and Sleep Research Society [1]

### Covariates

Covariates that are potential confounders or are likely to be associated with drop-out were selected based on previous literature and subject matter knowledge. As *baseline covariates* we included age (years) at baseline, sex, region of residence (Eastern, Southern, North/Central Europe), total breast feeding duration (months), pregnancy weight gain (kg), pregnancy smoking (never vs sometimes/daily), preterm birth (yes vs no), mother’s age at birth (years), family history of obesity (yes vs no), study region (control vs intervention region)^1^ [25, 26], maximum parental educational level (according to International Standard Classification of Education (ISCED) [27]), migrant status (no parent with migrant background vs at least one parent with migrant background) and baseline energy intake. In addition to the intervention variables described above, a well-being score (range: 12 for low to 48 for high well-being; only in analyses based on W0 to W2), maternal BMI and time since baseline (years) were used as *time-varying covariates* in the models. For the models based on accelerometer data, we further considered the valid wear time (min/day) and low-intensity physical activity (LPA; min/day) as time-varying covariates.

Details on outcome, exposure and covariate assessment are provided in Supplementary Material S1 (Additional File 1). Missing values were imputed as described in Supplementary Material S2 (Additional File 1).

### Causal contrast and statistical analysis

Our causal contrasts of interest were the 6-year and 13-year risks, in the sample population, of developing OW/OB if all individuals had adhered to the specified intervention strategies as opposed to following their natural behavior (i.e., observational analogue to per-protocol effect). These risks can be identified using the parametric g-formula, under standard causal assumptions detailed in Supplementary Material S3 (Additional File 1). The g-formula is the generalization of standardization for time-dependent exposures and confounders and has previously been used to estimate the effect of lifestyle interventions e.g. on the risk of adiposity or coronary heart disease [17, 28–30]. The parametric g-formula models the joint distribution of the covariates, exposures and outcomes over time in order to generate potential outcomes under different hypothetical interventions imposing specific behaviours, e.g. a specific maximum amount of daily screen time. More specifically, the joint distribution is typically modelled by a sequence of parametric regression models. The g-formula can be used to estimate the risk that would have been observed had all study subjects adhered to a given intervention strategy and none had been lost to follow-up. It can also be used to simulate a population under no intervention, the so-called natural course or ‘natural behavior’ strategy, i.e. children behave the way they would do when not being assigned to any hypothetical strategy. The methodology has been described in detail previously [12, 31].

### Implementation of the parametric g-formula

The basic steps are as follows: First, we fitted a sequence of regression models for all time-varying covariates and the exposure, and for OW/OB, using pooled person-time data. Next, we used these models to predict, by simulation, the counterfactual risk of OW/OB under each of the interventions. The latter is based on the following steps, carried out separately for each intervention strategy considered: (1) use the observed values of covariates at baseline; (2) predict the values of time-varying covariates and exposure at the next wave using the fitted parametric models; (3) ‘intervene’ by setting the values of the exposure to the values pre-determined by the given hypothetical intervention; (4) estimate the predicted risk of OW/OB using these new values; (5) repeat steps (2) through (4) for the entire study period (cf. Vangen-Lonne, Ueda [32]).

For modelling the outcome, i.e. OW/OB incidence, a pooled logistic regression was fitted to estimate the conditional discrete-time hazard at each follow-up time. Each time-varying predictor was classed as binary or continuous. Binary-dependent variables, like membership in a sports club, were modelled using logistic regression. Continuous variables were modelled using Tobit or truncated regression. To account for the high number of zero values, the consumption frequency of SSB was modelled based on both, a logistic model (using an indicator whether the covariate is > 0) to estimate the probability of the covariate being zero and a linear regression model for the natural log of the covariate restricted to records with the covariate being > 0 for the estimation of non-zero values.

All models included, as predictors, all baseline covariates and baseline values of the exposures, as well as the current and previous value of all binary and continuous covariates.

Domain expertise was used to define covariate order. Interactions were added to the models for the outcome and for the time-varying covariates with unsatisfactory model fit based on domain knowledge until the models reached a good fit.

As an analytic computation of the g-formula is only possible in very simple cases, Monte-Carlo simulation was used, where the sample size was set to the actual sample size (10 877 in the main analysis). To obtain confidence intervals 100 (nonparametric) bootstrap samples and corresponding percentiles were used.

We further estimated analogous causal contrasts for hypothetical interventions targeting only specific subgroups, i.e. children of parents with low/medium ISCED level or children of mothers who have a BMI > 25 kg/m^2^ as well as intervening only on males/females and younger vs older children (2 to < 6 years vs 6 to < 10 years at baseline).

In the subgroup of 2203 subjects with information on accelerometer-based MVPA (only W0 to W2), we additionally estimated the effect of hypothetical interventions on MVPA over a six-year time span.

Supplementary Material S4 (Tables S4a/b; Additional File 1) lists all baseline and time-varying covariates included as well as the functional form and type of model chosen for the covariates when being used as predictor/ response variable, respectively, in the main analyses (Table S4a) as well as in the model for MVPA (Table S4b) restricted to the subgroup with accelerometer data.

### Sensitivity analyses

In our study, time intervals between waves are rather long (up to 7 years) and measurements at the same wave are cross-sectional. We hence conducted two types of analyses: the first one allowed contemporaneous effects (e.g. screen time in W1 could act on OW/OB in W1 and so forth), i.e. we assumed each questionnaire to reflect exposure during the previous period (referred to as main model); the second analysis allowed only time-delayed effects to reduce the risk of reverse causation (e.g. screen time in W0, but not in W1, acts on OW/OB in W1 and so forth; presented as sensitivity analysis). These different modelling assumptions are visualized using directed acyclic graphs (DAGs) in Supplementary Material S5 (Additional File 1). As further sensitivity analyses, we a) estimated the effects over a 6-year (W0 to W2) instead of 13-year period (W0 to W3), b) included fruit juices in the calculation of SSB, c) added the distance to kindergarten/school/work as an additional time-varying covariate, and d) changed the ordering of time-varying covariates measured in the same wave when modelling the time-varying covariates.

All analyses were conducted using SAS 9.3 (Cary, NC). The SAS macro for application of the g-formula is available here [33].

## Results

Table 2 shows baseline characteristics of the 10 877 children that met the eligibility criteria of our main analysis. The mean age at baseline was 5.8 (standard deviation (SD): 1.9) years with 51.6% of the children being male. The observed risk of becoming OW/OB during the entire follow-up was 31.4%. Percentages of children observationally adhering to the different recommendations at W0 to W3 are displayed in Supplementary Material S6 (Additional File 1). While adherence to sleep (82.5% at W0 to 68.4% at W3), screen time (54.1% at W0 to 23.2% at W3) and SSB recommendations (82.0% at W0 to 57% at W3) declined with age, use of active transport increased (39.2% at W0 to 73.9% at W3).

**Table 2:** Baseline characteristics of the study sample comprising 10 877 children

		**N**	**%**
**Sex**	Male	5613	51.6
	Female	5264	48.4
**European region**	South	2454	22.6
	Middle	4932	45.3
	East	3491	32.1
**Educational level of parents**^a^	Low	678	6.2
	Medium	4808	44.2
	High	5391	49.6
**Maternal BMI**	≤ 25 kg/m^2^	7865	72.3
	> 25 kg/m^2^	3012	27.7
**Migrant status of parents**	Yes	1420	13.1
**Smoking during pregnancy**	Yes	1635	15.0
**Family history of obesity**	Yes	2260	20.8
**Preterm birth**	Yes	1270	11.7
		**Mean**	**SD**
**BMI z-score by Cole (2012)**		-0.1	0.8
**Age [years]**		5.8	1.9
**Follow-up time [years]**		3.2	4.2
**Duration of total breastfeeding [months]**		7.0	6.6
**BMI of mother (kg/m2)**		23.6	4.1
**Pregnancy—mother's age at birth of child [years]**		29.4	5.0
**Usual energy intake excluding misreports (kcal/day)**		1484	176
**Pregnancy—mother's gained weight [kg]**		14.0	5.5
**Well-being score**		40.2	4.5
**Screen time (hours/day)**		1.6	1.0
**Nocturnal sleep duration (hours/day)**		10.4	1.0
**SSB (times/week)**		2.6	5.5

Number and percentages for categorical variables; means and standard deviation (SD) for continuous variables

^a^Highest educational level of parents at baseline categorized according to the International Standard Classification of Education (ISCED) [27]

### Estimated effects of hypothetical interventions

When applying the parametric g-formula, the models in general performed well in estimating the risk factor distributions under no intervention (comparing the mean differences between the observed and simulated time-dependent variables). In addition, the model-based predicted risk of OW/OB over the 13-year period was close to the observed risk (30.7% vs 31.4%). This may serve as an indicator that the parametric models are not grossly misspecified.

Table 3 shows the 13-year risk of developing OW/OB under the different hypothetical behavioral interventions when intervening either on the whole study sample, only on children with low/medium parental ISCED or on children of mothers with BMI > 25 kg/m^2^. Table 4 presents the corresponding results for the subgroup with accelerometer data over a 6-year period.

**Table 3:** Population 13-year risk estimates of overweight/obesity using the g-formula

	Observed risk: 31.38	**Risk (%)**	**95% LCL**	**95% UCL**	**Population risk ratio**	**95% LCL**	**95% UCL**	**Population risk difference**	**95% LCL**	**95% UCL**	**Cumulative % intervened**^**a**^	**Average % intervened**^**b**^
**0**	**Natural course**	**30.7**	28.4	32.7	**1.00**	1.00	1.00	**0.00**	0.00	0.00	**0.0**	0.0
**1a**	**All subjects meet screen time recommendations**	**28.5**	25.5	30.6	**0.93**	0.86	0.98	**-2.21**	-4.39	-0.71	**97.1**	75.1
	Intervene only in subjects with low/medium parental ISCED	**30.1**	27.7	32.1	**0.98**	0.94	1.02	**-0.56**	-1.97	0.54	**49.3**	39.3
	Intervene only in subjects with mother's BMI > 25 kg/m2	**30.0**	27.9	32.0	**0.98**	0.95	0.99	**-0.71**	-1.47	-0.16	**27.0**	21.3
**1b**	**All subjects reduce screen time by 0.5 h/day**	**30.0**	27.9	32.1	**0.98**	0.96	0.99	**-0.64**	-1.16	-0.22	**97.1**	75.1
	Intervene only in subjects with low/medium parental ISCED	**30.5**	28.3	32.6	**0.99**	0.98	1	**-0.17**	-0.52	0.09	**49.3**	39.3
	Intervene only in subjects with mother's BMI > 25 kg/m2	**30.5**	28.2	32.5	**0.99**	0.99	1	**-0.2**	-0.36	-0.06	**27.0**	21.3
**2**	**All subjects are members in sports club**	**29.1**	26.5	31.0	**0.95**	0.91	0.99	**-1.58**	-2.73	-0.41	**77.1**	32.2
	Intervene only in subjects with low/medium parental ISCED	**29.6**	27.3	31.6	**0.97**	0.94	1	**-1.02**	-1.93	-0.06	**42.4**	19.2
	Intervene only in subjects with mother's BMI > 25 kg/m2	**30.0**	27.8	32.1	**0.98**	0.96	1	**-0.65**	-1.08	-0.08	**22.0**	9.5
**3**	**All subjects use an active form of transport**	**29.6**	26.9	31.7	**0.96**	0.93	1.02	**-1.12**	-2.17	0.53	**78.2**	36.0
	Intervene only in subjects with low/medium parental ISCED	**30.1**	27.7	32.3	**0.98**	0.96	1.02	**-0.53**	-1.36	0.67	**38.9**	17.9
	Intervene only in subjects with mother's BMI > 25 kg/m2	**30.3**	28.1	32.1	**0.99**	0.98	1.01	**-0.4**	-0.75	0.27	**21.5**	9.8
**4a**	**All subjects meet sleep recommendations**	**30.0**	27.5	31.9	**0.98**	0.96	0.99	**-0.64**	-1.07	-0.28	**58.5**	37.7
	Intervene only in subjects with low/medium parental ISCED	**30.3**	27.9	32.3	**0.99**	0.98	0.99	**-0.35**	-0.58	-0.15	**29.8**	19.4
	Intervene only in subjects with mother's BMI > 25 kg/m2	**30.4**	28.1	32.4	**0.99**	0.99	1	**-0.22**	-0.36	-0.1	**16.9**	10.8
**4b**	**All subjects increase sleep by 0.5 h**	**30.1**	27.6	32.0	**0.98**	0.97	0.99	**-0.59**	-0.94	-0.25	**58.5**	37.7
	Intervene only in subjects with low/medium parental ISCED	**30.4**	27.9	32.3	**0.99**	0.98	1	**-0.32**	-0.51	-0.13	**29.8**	19.4
	Intervene only in subjects with mother's BMI > 25 kg/m2	**30.5**	28.1	32.5	**0.99**	0.99	1	**-0.2**	-0.31	-0.09	**16.9**	10.8
**5**	**All subjects eat while doing something else non-daily**	**29.5**	26.8	31.5	**0.96**	0.91	1	**-1.22**	-2.72	-0.05	**70.5**	27.7
	Intervene only in subjects with low/medium parental ISCED	**29.9**	27.2	31.8	**0.97**	0.95	1	**-0.8**	-1.64	0.09	**37.9**	15.8
	Intervene only in subjects with mother's BMI > 25 kg/m2	**30.2**	27.7	32.3	**0.98**	0.97	1	**-0.48**	-0.95	-0.04	**19.7**	7.7
**6a**	**All subjects drink max 4 SSB per week**	**31.6**	29.2	33.5	**1.03**	1.01	1.06	**0.97**	0.18	1.82	**78.0**	46.4
	Intervene only in subjects with low/medium parental ISCED	**31.4**	28.9	33.4	**1.02**	1	1.04	**0.69**	0.15	1.25	**42.1**	26.5
	Intervene only in subjects with mother's BMI > 25 kg/m2	**31.0**	28.7	33.1	**1.01**	1	1.02	**0.36**	0.08	0.71	**22.6**	13.9
**6b**	**All subjects reduce SSB by 1/day**	**31.4**	29.0	33.4	**1.02**	1.01	1.05	**0.73**	0.16	1.4	**78.0**	46.4
	Intervene only in subjects with low/medium parental ISCED	**31.2**	28.8	33.2	**1.02**	1	1.03	**0.5**	0.12	0.93	**42.1**	26.5
	Intervene only in subjects with mother's BMI > 25 kg/m2	**30.9**	28.6	33.0	**1.01**	1	1.02	**0.27**	0.06	0.53	**22.6**	13.9
**7**	**All subjects adhere to recommendation for all variables (1a, 2, 3, 4a, 5 and 6a combined)**	**25.4**	22.1	27.7	**0.83**	0.74	0.9	**-5.27**	-7.84	-3.15	**100.0**	93.0
	Intervene only in subjects with low/medium parental ISCED	**28.3**	25.4	31.2	**0.92**	0.86	0.98	**-2.34**	-4.28	-0.64	**50.4**	47.7
	Intervene only in subjects with mother's BMI > 25 kg/m2	**28.7**	26.5	31.3	**0.94**	0.91	0.97	**-1.96**	-2.83	-1.05	**27.7**	26.0

Hypothetical interventions on entire cohort as well as on children of parents with low/medium ISCED level and children of mother’s with BMI > 25 kg/m^2^ at baseline using data from W0 to W3. Model allowing contemporaneous effects of exposures on the outcome

^a^The cumulative percent intervened on is the percent of the population required to change behavior in at least one wave

^b^The average percent intervened on is the average, across all waves, of the percent of the study population required to change behavior in a given wave

**Table 4:** Population 6-year risk estimates of overweight/obesity using the g-formula in subgroup of 2203 children with accelerometer data

Observed risk: 17.8	**Risk (%)**	**95% LCL**	**95% UCL**	**Population risk ratio**	**95% LCL**	**95% UCL**	**Population risk difference**	**95% LCL**	**95% UCL**	**Cumulative** **% intervened**^**a**^	**Average % intervened**^**b**^
**Natural course**	**18.1**	16.6	21.0	**1**	1	1	**0**	0	0	**0.0**	0.0
**All subjects perform at least 60 min MVPA**	**16.0**	14.3	19.3	**0.89**	0.81	0.96	**-2.06**	-3.7	-0.8	**88.3**	79.3
Intervene only in subjects with low/medium parental ISCED	**17.6**	16.0	20.6	**0.97**	0.93	1.01	**-0.50**	-1.35	0.25	**34.7**	30.8
Intervene only in subjects with mother's BMI > 25 kg/m2	**17.5**	15.6	20.1	**0.96**	0.92	0.99	**-0.65**	-1.45	-0.23	**20.0**	17.6
**All subjects not meeting recommendation increase MVPA by 15 min**	**16.6**	15.0	19.7	**0.92**	0.87	0.97	**-1.50**	-2.65	-0.6	**88.3**	79.3
Intervene only in subjects with low/medium parental ISCED	**17.7**	16.1	20.6	**0.98**	0.95	1.01	**-0.35**	-0.91	0.15	**34.7**	30.8
Intervene only in subjects with mother's BMI > 25 kg/m2	**17.6**	16.0	20.2	**0.97**	0.95	0.99	**-0.47**	-0.96	-0.16	**20.0**	17.6

Hypothetical interventions on entire subgroup as well as on children of parents with low/medium ISCED level and children of mother’s with BMI > 25 kg/m^2^ at baseline using data from W0 to W2. Model allowing contemporaneous effects of exposures on the outcome

^a^The cumulative percent intervened on is the percent of the population required to change behavior in at least one wave

^b^The average percent intervened on is the average, across all waves, of the percent of the study population required to change behavior in a given wave

**Fig. 2:**
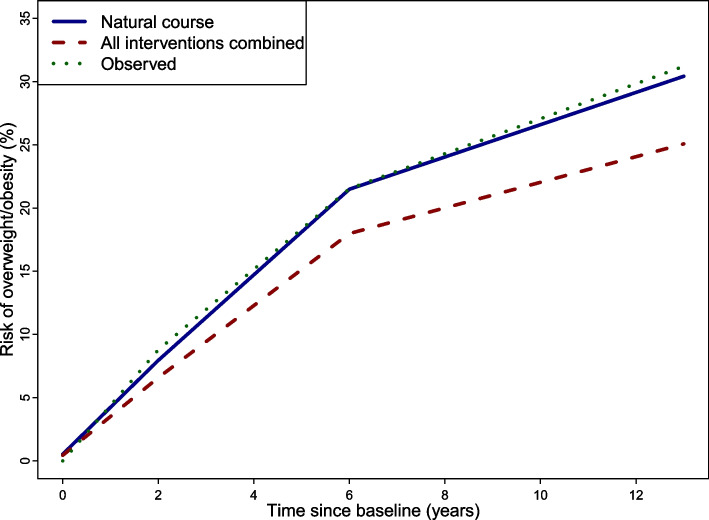
Risk plot depicting the 13-year risks of developing overweight/obesity (OW/OB) under no intervention (‘natural course’; blue line) and under the joint intervention (red line) estimated based on the parametric g-formula as well as the observed risk of developing OW/OB (green dotted line)

Among the lifestyle interventions, adhering to screen time (-2.21 [-4.39;-0.71]; risk difference (RD) and 95% bootstrap confidence interval (CI)) and MVPA recommendations (RD: -2.06 [-3.73;-0.80]) were the interventions with the largest risk differences concerning OW/OB incidence. This corresponds to a OW/OB risk reduction of 7% for meeting screen time over 13 years and 11% for meeting MVPA recommendations over 6 years. These were also the interventions with the highest number of participants intervened on, i.e. who would need to change their natural behavior (97.1% of the population for screen time, 88.3% for MVPA). Membership in a sports club (RD: -1.58 [-2.73;-0.41]), adherence to sleep recommendations (RD: -0.64 [-1.07;-0.28]) and ‘non-daily eating while doing something else’ (RD: -1.22 [-2.72;-0.05]) also showed small intervention effects. Unexpectedly, our results suggest that limiting SSB consumption increases the risk of developing OW/OB (RD: 0.97 [0.18;1.82]). The combined effect of all interventions based on recommendations (excluding MVPA as assessed only in a subsample) was a relative reduction of the OW/OB risk by 17% [95% CI: 10%;26%], i.e. from 30.7% to 25.4%, over the 13-year period (see Table 3 and Risk Plot in Fig. 2). According to the 95% CI, there was a relative reduction of the OW/OB risk by between 10 to 26% if all six specified behavioral interventions were followed.

### Results of shift interventions

Increasing sleep duration by 0.5 h had a similar effect on OW/OB incidence as compared to exact adherence to sleep recommendations (Table 3). A reduction of screen time by 0.5 h/day reduced the risk of developing OW/OB (RD: -0.64 [-1.16;-0.22]) by a lesser amount as compared to the effect of adherence to screen time recommendations. Increasing MVPA by as little as 15 min/day over a six-year period reduced the risk of OW/OB by 1.5 percentage points (Table 4).

### Interventions targeting specific vulnerable groups

**Fig. 3:**
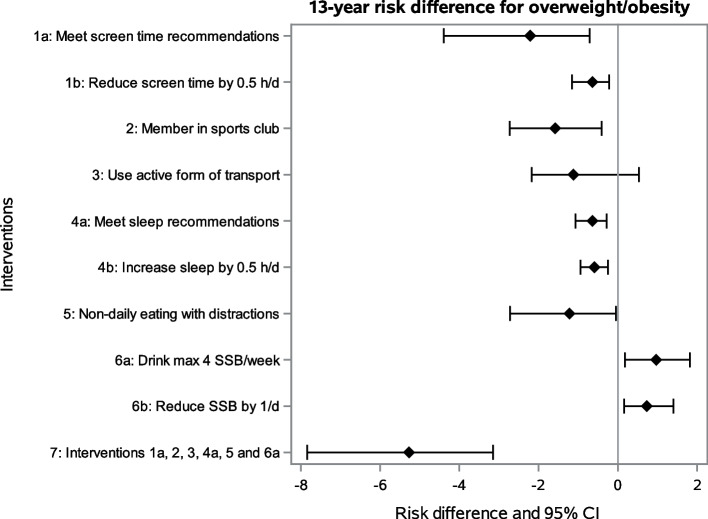
Population risk differences and 95% confidence intervals using the g-formula; hypothetical interventions on entire cohort using data from W0 to W3. Model allowing contemporaneous effects of exposures on the outcome

Interventions targeting children of parents with low/medium educational level or children of mothers with BMI > 25 kg/m^2^ have smaller intervention effects on population level while requiring an intervention on a much smaller number of children (see e.g. ‘Average % intervened on’ in Table 3). The most effective intervention in children of parents with low/medium educational level was being a member in a sports club; for children of mothers with BMI > 25 kg/m^2^ meeting screen time recommendations, meeting MVPA recommendations and membership in a sports club exhibited the largest intervention effects (see Tables 3 and 4, Fig. 3, and Supplementary Material S7 (Additional File 1)).

### Results when intervening only in subgroups by age and sex

Interventions on screen time showed larger effects in girls as compared to boys while the opposite was observed for membership in a sports club and ‘non-daily eating while doing something else’ (Supplementary Material S8, Tables S8a and S8b, Figure S8c; Additional File 1). Also, when comparing the effects among age groups (‘2 to < 6 years’ vs ‘6 to < 10 years’ at baseline), few differences were found, with the effect of adherence to screen time recommendations being larger in the younger age group (Supplementary Material S8, Tables S8d and S8e, Figure S8f; Additional File 1). Being member in a sports club had a larger intervention effect in the older age group.

### Sensitivity analyses

When allowing only for time-delayed effects between the exposures and OW/OB, the estimated effects of the hypothetical interventions moved slightly towards the null for all exposures except screen time, and the unexpected harmful effect of limiting SSB consumption became a null effect (see Supplementary Table S9a and Figure S9b; Additional File 1). When restricting our main model to data from W0 to W2, i.e. to a 6-year period, estimated effects again shrunk slightly towards the null (see Supplementary Table S10a and Figure S10b; Additional File 1). The unexpected harmful effect of limiting SSB consumption on OW/OB increased when including fruit juices in the calculation of SSB (RD: 1.51 [0.35;2.58] for SSB including fruit juices and 0.97 [0.18;1.82] for SSB excluding fruit juices). When adding the distance to kindergarten/school/work as an additional time-varying covariate to our main model, the intervention effects of active transport ([RD: 1.99 [-3.80;-0.29]) as well as of the joint intervention (RD: 6.02 [-9.01;-3.82]) increased while the effects of the other single interventions remained similar as compared to the main analysis (see Supplementary Material S11; Additional File 1). Under alternative arbitrary orderings of the covariates, estimated risks changed moderately without altering the overall interpretation of results.

## Discussion

Our study estimates the causal effects of hypothetical interventions imposing full adherence to recommendations, or shifts in behaviors, on the 13-year risk of developing OW/OB from childhood to adolescence using observational data. Our results were compatible with a relative reduction of the OW/OB risk by 10 to 26% when adhering to all six behavioral interventions as compared to no intervention. While in a group of 100 children about 31 [28.4; 32.7] children would develop OW/OB without intervention, about 25 [22.1; 27.7] would develop OW/OB under the joint intervention suggesting that incidental OW/OB could be prevented in about 5 to 6 children. In our sample, almost no child observationally adhered to all behavioral guidelines. This indicates that interventions targeting only children from vulnerable groups may not be sufficient.

The most effective single interventions were meeting screen time and MVPA recommendations. For instance, complying with the MVPA recommendation alone resulted in a relative reduction of the OW/OB risk by 4 to 19% over a six-year period. The strength of the other single intervention effects on the risk of OW/OB was modest and showed relative reductions of the OW/OB risk of around 2 to 4%. The interventions on meeting sleep recommendations had only small effects which may be due to the fact that a large proportion of participants already adhered to sleep recommendations.

The relative reduction of the OW/OB risk of 10 to 26% under the joint intervention imposing continual adherence to all recommendations may be considered moderate. However, even in real-life interventions only modest effectiveness or mixed results of programs targeting a healthy lifestyle including dietary behavior and/or physical activity on the OW/OB incidence or prevalence rates were reported [34–37]. In those studies, intervention periods were much shorter (up to 3 years) compared to our hypothetical intervention which was sustained (i.e. with continued adherence) over a period of 13 years.

Numerous other behavioral intervention studies focused on BMI changes and results were synthesized in systematic reviews. They mostly reported risk reductions but also mainly small effects, e.g. of school-based interventions with parental involvement intervening on physical, sedentary and dietary behavior [9] or interventions on PA and/or diet [10, 11, 38].

In a previous hypothetical intervention targeting dietary behavior, screen time and MVPA in 11-year-old children, the population mean difference in BMI after two years under the combined interventions compared to no intervention was -0.28 kg/m^2^ (95% CI -0.59; 0.07). In contrast to our results, none of the individual interventions had an effect on BMI [16]. Also a 23-months hypothetical intervention in 1 to 5 year old children found little or no effects of single behavioral interventions targeting e.g. SSB, TV time and playing on the playground daily on weight-for-height z-score. Only interventions on breast feeding had a protective effect [17]. In a recent simulation study, the effect of single and combined behavioral micro-level interventions (e.g. home-visit programs) as well as macro-level interventions (e.g. policies on food labelling, healthy menu in restaurants, etc.) was investigated. Small or no beneficial effects of behavioral interventions were reported; only interventions that targeted breastfeeding resulted in a moderate effect on BMI z-score [18].

The lower effectiveness of PA interventions in real-life intervention trials [10] may be due to the shorter duration of interventions, the frequent reliance on self-reported data as well as due to the hypothetical full adherence to MVPA recommendations imposed in our study whereas full adherences may be unrealistic in real-life interventions.

In contrast to previous studies [24], we observed an increased risk of OW/OB when limiting SSB consumption. The unexpected effect in our study might be due to reverse causation as the effect disappeared when allowing only time-delayed effects of exposures on the risk of OW/OB in a sensitivity analysis.

Besides their effectiveness, the practical feasibility of interventions is an important requirement which was addressed in our study by also estimating the effects of shifting behaviors towards the recommendations. For children it may be easier to achieve small behavioral changes like increasing MVPA by 15 min/day or sleep duration by 30 min/day compared to strict adherence to recommendations. Our study shows a similar effectiveness of the shift interventions for MVPA and sleep suggesting that even small behavioral changes can have a beneficial effect on OW/OB. In practice, increasing sleep duration, for instance, may be achieved by setting a regular bedtime routine since going to bed earlier and increasing the time in bed can increase the overall sleep duration [39, 40]. Limiting screen time by parental rules may be feasible in young children but more difficult to impose on adolescents who show a higher degree of autonomy and typically own a smartphone, tablet and/or computer [41–43]. Parents should also restrict TV watching, smartphone use or exposure of their children to other distractors during meals. Adherence to MVPA recommendations can possibly be encouraged by active commuting, by a sports club membership and by increasing the time for school sports. Active commuting can be promoted by creating environments with safe walking and cycling lanes and by facilitating the allocation of a kindergarten/school/work close to home [44]. However, the success of interventions to increase and sustain PA depends on encouragement, role modelling and support by parents as well as by peers and teachers [45–48]. Increasing the time for school sport may be the most feasible way since school hours have been shown to contribute 55%, 43% and 46% to total daily sedentary, light PA and MVPA time compared to only 37%, 29% and 23% during leisure time, respectively [49].

We further estimated the population intervention effects when intervening only on certain vulnerable groups which may be of particular interest for policy makers. For most interventions we did not observe stronger effects relative to the number of children in the vulnerable groups. This may be explained by the design of the interventions which were only targeted at children not ‘naturally’ adhering to a recommended behavior. As reported previously [50, 51], children from vulnerable groups typically show more unhealthy behaviors so that in consequence interventions at population level will mainly apply to children from vulnerable groups. However, our results suggest that membership in a sports club, in particular, may be a promising intervention target for children of parents with low/medium education as well as for children of mother’s with OW/OB. Low-threshold access could be enabled by subsidizing sports club memberships for children with low educational or socioeconomic background.

Regarding sex-specific differences we observed a stronger effect of the joint interventions in boys than in girls which resulted mainly from a stronger intervention effect of membership in a sports club and of ‘non-daily eating while doing something else’ in boys. In contrast, girls appear to benefit more from a screen time reduction. Studies have shown that meal time distractions like watching TV while eating are associated with less healthy food habits and childhood obesity [52, 53] whereas studies under laboratory conditions did not observe an effect of such distractors on caloric intake in children and adolescents [54]. In the HELENA study, adolescent girls watching TV > 2 h/day had a higher frequency of eating food during TV viewing than boys. Additionally, more unhealthy foods were consumed in adolescents watching TV > 2 h/day than in those with less daily TV time [55]. Thus, particularly unhealthier food choices and increased sedentary time [56] may explain why high screen time and ‘eating while doing something else’ contribute to excessive weight gain, and interventions on both are likely to reduce the risk of developing OW/OB.

### Strengths and limitations

The g-formula allowed us to estimate risks of meaningful long-term intervention strategies and is more stable and efficient than inverse probability weighting albeit at the price of more modelling assumptions (see Supplementary Material S4; Additional File 1) and computational effort [57]. Lifestyle data were collected 2, 6, and 13 years from baseline rather than e.g. weekly. Therefore, we had to assume that each data wave is a good summary of the average behavior during the previous period. We do not expect that this assumption will hold exactly; typically, lifestyle behaviors will not remain constant for periods up to 7 years. However, we expect that the richness of our observational data allows us to approximately characterize children’s behaviors over 13 years and to adjust for much confounding. Moreover, by including contemporary covariates for confounder adjustment we indirectly adjust for potential unmeasured confounding factors that occur between waves [31]. A certain degree of measurement error is expected as all behavioral factors except for MVPA were proxy- or self-reported and especially dietary data are prone to misreporting which may have contributed to bias. Our aim was to consider a combination of behavioral interventions that can easily be translated into health recommendations. Hence, we included only a single dietary component, SSB, instead of e.g. deriving dietary patterns or complex food scores which are in general difficult to interpret. It should further be noted that being a member in a sports club does not automatically imply being physically active. However, previous research indicated that sports club members are more likely to reach MVPA recommendations [58]. Additionally, being a sports club member is positively associated with objectively measured time spent in vigorous [58] and MVPA [49, 56] and with a higher parental educational attainment [59, 60]. Our cohort suffers from a large degree of drop-out over the 13-year time span. Attrition in the IDEFICS cohort was found to be associated with a higher weight status of children, older age, lower parental education, and parent's migration background [61]. The g-formula simulates the risk under no drop-out if all important factors predictive of drop-out are included. We included a large number of covariates but still cannot preclude that selection effects may have affected our results. The IDEFICS/I.Family study sample was not completely random due to cost restrictions and for feasibility reasons. The proportion of children with well-educated parents is higher in our sample compared with the general population. These shortcomings may hence limit the generalizability of the results. Due to the fact that participants are likely to give socially desirable answers and due to selection towards a healthier, well-educated population, our effect estimates may be underestimated.

Strengths of our study include the multi-center nature of the study including several countries, the standardized and highly quality controlled assessment procedures, the large study sample and long follow-up time. To the author’s knowledge, this is the first study demonstrating the effectiveness of various hypothetical behavioral interventions in children over long time spans comparing shift interventions in addition to interventions imposing sustained adherence to recommendations. The focus on certain vulnerable subgroups is a further unique aspect of this study.

## Conclusions

In a group of 100 children, about 31 children would develop OW/OB over a 13-year period without intervention and about 25 when adhering to all six behavioral interventions. This suggests that incidental OW/OB could be prevented in about 5 to 6 children under a joint intervention which corresponds to a relative reduction of the risk of developing OW/OB by between 10 to 26%. Meeting MVPA and screen time recommendations had the largest intervention effects. However, relative to the number of children intervened on, sports club membership and ‘non-daily eating while doing something else’ were most effective. In addition, the results of the shift interventions for MVPA and sleep suggest that even small behavioral changes can have a beneficial effect on OW/OB risk. Policies should promote sports activities, e.g. by enabling low-threshold access to different PA options and creating environments with safe walking and cycling routes. Providing sports club memberships or similar offers may help to increase PA in vulnerable groups.

Nevertheless, even when implementing the joint hypothetical interventions, the absolute risk of developing OW/OB remained at a rather high level of 25.4%. Further strategies are required to prevent OW/OB such as policies and interventions promoting a healthy food choice, improving the psychosocial well-being of children and supporting families to create a healthy environment for children.

### Supplementary Information

**Additional file 1:** **S1.** Description of outcome, exposure and covariate assessment; **S2.** Imputation of missing values; **S3.** Identifying assumptions and their plausibility; **S4.** Functional form and type of model chosen for the covariates when being used as predictor/ response variable in the 6-year and 13-year analyses; **S5.** Figure depicting assumed time-order between covariates, exposures and outcome in model allowing contemporaneous effects and in model allowing only time-delayed effects; **S6.** Numbers and percentages of children adhering to the different recommendations at W0 to W3; **S7.** Graphical display of population risk differences when intervening only on children of mother’s with BMI > 25 kg/m^2^ and children of parents with low/medium ISCED level; **S8.** Intervention effects when intervening only in males/females or younger/older children (Tables and Figures); **S9.** and **S10.** Results of sensitivity analyses (Tables and Figures); **S11.** Sensitivity analysis adding the distance to kindergarten/school/work as a time-varying covariate.

## Data Availability

Due to the sensitive nature of data collected, ethical restrictions prohibit the authors from making the minimal data set publicly available. Each cohort center received approval of the corresponding local Ethical Commission and participants did not provide consent for data sharing. Data are available on request and all requests need approval by the study’s Steering Committee. Interested researchers can contact the study coordinator (ahrens@leibniz-bips.de) to request data access. All requests for accessing data of the IDEFICS/I.Family cohort are discussed on a case-by-case basis by the Steering Committee.

## Abbreviations

BMI: Body mass index
CI: Confidence interval
ISCED: International Standard Classification of Education
MVPA: Moderate-to-vigorous physical activity
OW/OB: Overweight/obesity
PA: Physical activity
RCT: Randomized controlled trial
RD: Risk difference
SD: Standard deviation
SSB: Sugar-sweetened beverages

## Footnotes

1 The IDEFICS study was designed as a controlled intervention study. Although the expected intervention effects on behavioral factors as well as on the prevalence of overweight and obesity could not be shown in the target population as a whole, we adjusted for the corresponding study regions to account for potential effects the IDEFICS intervention may have had on behavioral factors in individual countries.
