# Supplementary material for: The effects of hypothetical behavioral interventions on the 13-year incidence of overweight/obesity in children and adolescents
Source: Int J Behav Nutr Phys Act. 2023 Aug 24;20:100. doi: 10.1186/s12966-023-01501-6 (PMC10463721; doi:10.1186/s12966-023-01501-6)
Supplement: Supplementary file 1 — Additional file 1: S1. Description of outcome, exposure and covariate assessment; S2. Imputation of missing values; S3. Identifying assumptions and their plausibility; S4. Functional form and type of model chosen for the covariates when being used as predictor/ response variable in the 6-year and 13-year analyses; S5. Figure depicting assumed time-order between covariates, exposures and outcome in model allowing contemporaneous effects and in model allowing only time-delayed effects; S6. Numbers and percentages of children adhering to the different recommendations at W0 to W3; S7. Graphical display of population risk differences when intervening only on children of mother’s with BMI > 25 kg/m2 and children of parents with low/medium ISCED level; S8. Intervention effects when intervening only in males/females or younger/older children (Tables and Figures); S9. and S10. Results of sensitivity analyses (Tables and Figures); S11. Sensitivity analysis adding the distance to kindergarten/school/work as a time-varying covariate. [file 12966_2023_1501_MOESM1_ESM.pdf]

Supplementary Material S1: Description of outcome, exposure and covariate assessment

Outcome

Body height was measured with a calibrated stadiometer (Seca 225/213 stadiometer, Birmingham, UK) to the nearest 0.1 cm. Weight was assessed on a calibrated Tanita scale (Tanita Europe GmbH, Sindelfingen, Germany) accurate to 0.1 kg in a fasting state with children wearing light underwear. In W3, body height and weight were self-reported by the study participants. Weight (kg) divided by height (m) squared was calculated to determine the Body Mass Index (BMI). BMI was categorized according to the extended IOTF criteria for study subjects <18 years [1] and according to WHO for subjects ≥18 years [2].

Exposures and covariates

Unless otherwise stated, exposure/covariate information was obtained from parentally reported questionnaires or self-reported questionnaires (in children/teens ≥ 12 years) completed at the W0, W1, W2 and W3 surveys. Questions on pregnancy-related variables were posed to biological mothers only. Several variables were dichotomized in order to reduce model complexity considering the large number of included variables.

Non-modifiable factors

Age (in years) at baseline, sex (female; ref male), region of residence (North/Central Europe including Belgium, Sweden and Germany, Southern Europe including Cyprus, Italy, Spain and Eastern Europe including Estonia and Hungary), family history of obesity (diagnosed obesity in at least one parent, grandparent or sibling; yes vs no), study region (control vs intervention region) and migrant status (at least one parent born in foreign country vs both parents born in the country of residence) were considered as non-modifiable covariates in the analyses.

Educational level of parents

Highest educational level of parents at baseline was categorized according to the International Standard Classification of Education (ISCED).[3] The maximum ISCED level of both parents was categorized into three categories (low=ISCED levels 0,1,2, medium= ISCD levels 3,4,5 vs high=ISCED levels 6,7,8).

Early life factors

For early life factors, information was assessed in W0 via questionnaires and updated by W1 values in case of missing W0 data.

Maternal body mass index (BMI; kg/m2): Maternal BMI at W0, W1 and W2 was calculated as weight (kg) divided by height (m) squared where weight/height were self-reported in W0 and W1 and measured in W2. In W3 no information on maternal BMI is available.

Smoking of mother during pregnancy: For smoking frequency answer categories ranged from 'Never', 'Rarely, at max once a month', 'Several occasions a week' to 'Daily'. To improve stability of model

estimates, the original smoking categories were dichotomized into ‘Never’ vs. ‘Rarely/at least several occasions a week/daily’.

Weight gain during pregnancy (kg): Information was obtained from biological mothers via questionnaires.

Gestational age of new-born at delivery: A binary indicator was constructed for children delivered at term vs. children born pre-term (≤ 37th gestational week; yes vs no), information was obtained from biological mothers via questionnaires.

Birth weight (g) of the child and mother’s age at birth (years) was reported by mothers.

Total breastfeeding duration: Starting and ending months of exclusive breastfeeding and breastfeeding combinations with solid foods and/or formula milk were used to derive the total breastfeeding duration.

Well-being

Well-being score: Psychosocial well-being was measured with 16 items of four subscales of the “KINDL-R Questionnaire for Measuring Health-Related Quality of Life (HRQoL) in Children and Adolescents” (emotional well-being, self-esteem, family life and relations to friends).[4, 5] At W2, response categories corresponded to the original 5-point Likert scale (never, seldom, sometimes, often, all the time). At W0 and W1 the two highest response categories were combined into one category. Therefore, we deviated from the original scoring (1-5 points per item) and assigned 0 points for “Never” and 3 points for both “Often” and “All the time” (at follow-up) or “Often/All the time” (at baseline), respectively (six negatively worded items were coded reversely). Consequently, the score ranged from 0-48 with a higher score indicating a higher well-being. As the instruments changed in W3 in study participants ≥ 18 years, the well-being score was only calculated for waves W0 to W2.

Lifestyle exposures

Nocturnal sleep duration (hours/night): At W0, information on sleep duration was collected in the context of a standardized 24-h recall. Next to questions on dietary intakes, parents were asked about their child’s get up time in the morning as well as bed time (hour/minute) of the previous day. Nocturnal sleep duration was calculated as difference between bed time and get up time resulting in a continuous measure of sleep hours per night as described previously [6]. At W1 and W2, participants reported sleep duration in hours and minutes in self-completion questionnaires, i.e. the instructions read as follows: “What is the amount of time the child sleeps during a 24-hour period on weekdays? Give separate information for night time sleep and naps in the daytime.” Analogously, information was collected for weekend days/vacations. The weighted average of nocturnal sleep duration was calculated as follows: (nocturnal sleep duration on weekdays*5 + nocturnal sleep duration on weekend days*2) / 7. Reported usual sleep duration of < 5 hours/night or > 15 hours/night were considered implausible and set to missing.

Average screen time (hours/week): The time spent with audiovisual media was calculated based on the reported hours/minutes watching TV/video/DVD and hours/minutes sitting in front of a computer/game

console. In W2 and W3, additional information on web-based screen time like streaming movies was considered. A weighted average over weekdays and weekend days was calculated.

Moderate to vigorous physical activity (MVPA; hours per day): Habitual PA was assessed using Actigraph accelerometers (Actigraph,LLC, Pensacola, FL, USA). In W0 and W1, either ActiTrainer or GT1M monitors were used, while in W2 either GT1M or GT3x + devices were used. Participants were asked to wear the accelerometers for at least 3 days (including 1 weekend day) at W0 and W1 and for 7 days at W2. Accelerometers were mounted on the right hip during waking hours of each child using an elastic belt adjusted to ensure close contact with the body. Details on processing of accelerometer data in the IDEFICS study as well as first descriptive results of accelerometer data of the IDEFICS study can be found in Konstabel et al. [7] Valid measurements were defined as recording more than 360 min of at least one weekday and one weekend day after exclusion of non-wear time according to Choi et al. [8] Non-wear time was identified using a 60 min window for each epoch to detect 30 min consecutive zero counts allowing breaks of 2 min of non-zeros. The threshold for valid measurements of at least 360 min for at least one weekday and one weekend day was chosen as a trade-off between accuracy and sample size and is discussed in Konstabel et al. [7] Before assigning intensity ranges, we here used a penalized expectile regression to smoothen the accelerometer counts that has been recently proposed in Wirsik et al. [9] MVPA and low physical activity (LPA) in minutes per day were then derived based on Evenson cut-off points for smoothed counts per minute (light: 104–2295, moderate: 2296–4011 cpm, vigorous: > 4011 cpm) [10]. Due to the compositional nature of the PA data, our models were adjusted for LPA when estimating the effects of interventions on MVPA on the incidence of OW/OB.

Membership in sports club (yes vs no): A variable indicating whether the child was member in a sports club was used as a proxy for physical activity.

Active transport (yes vs no): Active transport to/from kindergarten or school was considered in case the child usually gets to and from kindergarten/school either walking or cycling in W0, W1 and W2. In W3, times spend commuting to work/school and back home by walking, cycling, public transport, car/taxi or other transport modes were reported. If the times spend walking or cycling to work/school and back were greater than zero, an active form of transport was assumed.

Dietary variables

The following categorical variable was used to reflect the family meals: “Frequency of child eating while doing something else, e.g. watching TV, playing, sitting at a computer, looking at a book” (1=”Never or rarely”, 2=”Several times per week”, 3=”Once a day”, 4=”On several occasions per day”). Categories 1 and 2 as well as categories 3 and 4 were merged to derive a dichotomous variable with categories “Daily” vs “Non-daily”.

In W0, W1, W2 and W3 information on food frequencies were assessed based on the so-called Children’s Eating Habits Questionnaire–food frequency section (CEHQ-FFQ). The CEHQ-FFQ was designed as a screening tool to assess eating behaviors associated with overweight, obesity and general health in children. It covered 43 food times in W0, 46 in W1 and 58 in W2 with the following answer categories: ‘never/less than once a week’, ‘1–3 times a week’, ‘4–6 times a week’, ‘1 time per day’, ‘2 times per day’, ‘3 times per day’, ‘4 or more times per day’ and in W1 and W2 ‘I have no idea’.

These categories were converted into times per week ranging from 0 up to 30. Based on the converted food consumption frequencies, we calculated the following variables that were used in the present analyses:

- *Sugar-sweetened beverages (times/day):* In W0 and W1, sweetened drinks including sports drinks, bottled or canned tea, syrup-based drinks and similar; in W2 and W3 additionally carbonated sugar sweetened drinks, sweetened coffee and sweetened tea. In sensitivity analysis, we also included fruit juices in the calculation of SSB consumption frequencies. Reported consumption frequencies of > 8 times/day were considered implausible and set to missing.

Supplementary Material S2

Imputation of missing values

For the imputation, data were arranged in wide format, i.e. a new variable is built for each repeated measurement (W0, W1, W2, W3). Then standard FCS (fully conditional specification) imputation was performed once. All outcomes and exposures at the different assessment waves used in the final analyses were included in the procedure. The percentages of missing values ranged from 0% (age, sex) up to 49.5% (energy intake at W0) depending on the variable considered. For the majority of variables less than 5% of the values were missing. The outcome did not contain any missing values. Data were imputed sequentially for each wave. The number of missing values for all variables are presented in Supplementary Table S2 below.

MI combined with bootstrap is challenging and computationally intense such that we performed only a single imputation. We acknowledge that the calculation of the bootstrap confidence intervals (in course of the g-formula calculations) do not account for the uncertainty in the imputation.

Variable	Label	N	Number missing values
**Wave 0 (N=10877)**			
**id**	**ID-Number**	10877	0
**country**	**Country**	10877	0
**sex**	**Sex of the child**	10877	0
**contr_interv**	**Study group (Control or Intervention)**	10877	0
**hist_obes**	**Family history of obesity (yes/no)**	9468	1409
**bf_total**	**Duration of total breastfeeding [months]**	10356	521
**age_birth**	**Pregnancy - mother's age at birth of child [years]**	10447	430
**preg_w_up**	**Pregnancy - mother's gained weight [kg]**	9949	928
**birth_w**	**Birth - child's weight [g]**	10596	281
**migrant**	**Migrant status of parents**	10772	105
**smoke_preg**	**Smoking during pregnancy**	10197	680
**preterm**	**Preterm birth (yes/no)**	10709	168
**wb_score**	**Well-being score**	10103	774
**time**	**Assessment wave**	10877	0
**eatdoelse**	**Child eating and doing something else**	10747	130
**water**	**Water consumption frequency**	10300	577
**SSB2**	**Sugar-sweetened beverages (excluding fruits juices)**	10813	64
**SSB**	**Sugar-sweetened beverages (including fruits juices)**	10633	244
**sleep_night**	**Nocturnal sleep duration (hours)**	5939	4938
**age**	**Age [years]**	10877	0
**bmi**	**Body Mass Index**	10877	0
**bmi_cat_cole_12**	**BMI in categories by Cole (2012)**	10877	0
**bmi_score_cole_12**	**z-score of BMI by Cole (2012)**	10877	0
**screen_time**	**Screen time (hours/day)**	10551	326
**isced_cat2011**	**ISCED-Category 2011: Max of both parents**	10806	71
**club_mbr**	**Leisure - child member in sports club**	10544	333
**act_transport**	**Active transport (yes/no)**	10633	244
**bmi_m**	**BMI of mother (kg/m2)**	10530	347
**energy_ex_MR**	**Usual energy intake excluding misreports (kcal/day)**	6482	4395
**Wave 1 (N=6871)**			
**wb_score_t1**	**Well-being score**	6496	375
**eatdoelse_t1**	**Child eating and doing something else**	6811	60
**water_t1**	**Water consumption frequency**	6556	315

**SSB2_t1**	**Sugar-sweetened beverages (excluding fruits juices)**	6795	76
**SSB_t1**	**Sugar-sweetened beverages (including fruits juices)**	6699	172
**sleep_night_t1**	**Nocturnal sleep duration (hours)**	5979	892
**age_t1**	**Age [years]**	6871	0
**bmi_t1**	**Body Mass Index**	6871	0
**bmi_cat_cole_12_t1**	**BMI in categories by Cole (2012)**	6871	0
**bmi_score_cole_12_t1**	**z-score of BMI by Cole (2012)**	6871	0
**screen_time_t1**	**Screen time (hours/day)**	6683	188
**isced_cat2011_t1**	**ISCED-Category 2011: Max of both parents**	6775	96
**club_mbr_t1**	**Leisure - child member in sports club**	6673	198
**act_transport_t1**	**Active transport (yes/no)**	6728	143
**bmi_m_t1**	**BMI of mother (kg/m2)**	6596	275
**Wave 2 (N=3023)**			
**wb_score_t3**	**Well-being score**	2866	157
**eatdoelse_t3**	**Child eating and doing something else**	2996	27
**water_t3**	**Water consumption frequency**	3006	17
**SSB2_t3**	**Sugar-sweetened beverages (excluding fruits juices)**	3017	6
**SSB_t3**	**Sugar-sweetened beverages (including fruits juices)**	2994	29
**sleep_night_t3**	**Nocturnal sleep duration (hours)**	2941	82
**age_t3**	**Age [years]**	3023	0
**bmi_t3**	**Body Mass Index**	3023	0
**bmi_cat_cole_12_t3**	**BMI in categories by Cole (2012)**	3023	0
**bmi_score_cole_12_t3**	**z-score of BMI by Cole (2012)**	3023	0
**screen_time_t3**	**Screen time (hours/day)**	2618	405
**isced_cat2011_t3**	**ISCED-Category 2011: Max of both parents**	3010	13
**club_mbr_t3**	**Leisure - child member in sports club**	3003	20
**act_transport_t3**	**Active transport (yes/no)**	2967	56
**bmi_m_t3**	**BMI of mother (kg/m2)**	2253	770
**Wave 3 (N=1466)**			
**eatdoelse_t5**	**Child eating and doing something else**	1462	4
**water_t5**	**Water consumption frequency**	1458	8
**SSB2_t5**	**Sugar-sweetened beverages (excluding fruits juices)**	1461	5
**SSB_t5**	**Sugar-sweetened beverages (including fruits juices)**	1452	14
**sleep_night_t5**	**Nocturnal sleep duration (hours)**	1450	16
**age_t5**	**Age [years]**	1466	0
**bmi_t5**	**Body Mass Index**	1454	12
**bmi_cat_cole_12_t5**	**BMI in categories by Cole (2012)**	1454	12
**bmi_score_cole_12_t5**	**z-score of BMI by Cole (2012)**	1454	12
**screen_time_t5**	**Screen time (hours/day)**	1296	170
**club_mbr_t5**	**Leisure - child member in sports club**	1459	7
**act_transport_t5**	**Active transport (yes/no)**	1336	130

Supplementary Table 2: Number of missing values for all variables at the different assessment waves

Supplementary Material S3: Identifying assumptions and their plausibility

Conditional and sequential exchangeability: This assumption demands that the potential outcomes under certain fixed exposure levels are independent of the observed exposures. It is made within levels of past observed covariate values (conditional) and at each time point (sequentially). Informally, we speak of "no unmeasured confounding". As a common issue in observational data, unmeasured confounding cannot be fully excluded e.g. due to lack of genetic information. However, we adjusted for many potential (time-dependent) confounders and considered proxy indicators for missing confounders like e.g. the family history of obesity for genetic factors.

Causal consistency: This assumption is fulfilled when the treatment strategies being assessed are well-defined and correspond to the treatment strategies observed in the data, e.g. the outcome for a subject who happens to adhere to sleep time recommendations is the same as if he/she had been assigned to adhere to sleep time recommendations in the target trial, which is plausible.

Positivity: The validity of the results relies on the assumption of positivity, which requires that all intervention strategies should be observed within joint cross-classification of all confounders. The positivity assumption was checked empirically. Random non-positivity seems a potential concern for our causal analysis as there are only few subjects who observationally adhered to the joint interventions. By using the g-formula, we implicitly accept the model-based counterfactual extrapolation for covariate-exposure combinations where data are sparse. However, under correct models, the parametric g-formula is less prone to bias induced by positivity violations as compared to e.g. inverse probability weighting [11]. In our study, we conducted a sensitivity analysis using inverse probability of censoring weighting (IPCW) instead of the g-formula for estimating the risk under the natural course from the observed data and did not find substantial differences. When comparing our g-formula estimates under the natural course with those obtained based on inverse probability of censoring weighting (IPCW), less than 1% of our study population obtained a large weight (weights up to 1865) suggesting that violation of positivity is a problem in less than 1% of our population.

Correct model specification: The g-formula requires correct specification of the conditional (on the past) probabilities of the outcome and time-varying covariates in all follow-up intervals. Due to the use of multiple models, the parametric g-formula is especially vulnerable to the assumption of correct model specification. Informal checking is possible by comparison of the

observed data to the data simulated under the natural course. We compared the observed means of the outcome and time-varying covariates with those predicted by our models. The parametric g-formula closely replicated the observed risk and the mean covariates under the natural course.

Supplementary Material S4: Tables S4a and S4b

Variable	Purpose	Time period of variable assessment	Type of model when used as dependent variable	Functional form when used as predictor	Modelling type selected in SAS macro[#] for covariate history (covXptype)
**Baseline**					
Age at cohort entry	Confounder	Baseline	Not predicted	Continuous	
Age squared					
Age cubic					
Sex	Confounder	Baseline	Not predicted	Binary	
Region of residence	Confounder	Baseline	Not predicted	Categorical (3 categories)	
Parental educational level	Confounder	Baseline	Not predicted	Categorical (3 categories)	
Migrant status	Confounder	Baseline	Not predicted	Binary	
Pregnancy weight gain	Confounder	Baseline	Not predicted	Continuous	
Maternal age at birth	Confounder	Baseline	Not predicted	Continuous	
Indicator for preterm birth	Confounder	Baseline	Not predicted	Binary	
Smoking during pregnancy	Confounder	Baseline	Not predicted	Binary	
Total breast feeding duration	Confounder	Baseline	Not predicted	Continuous	
Baseline energy intake	Confounder	Baseline	Not predicted	Continuous	
Family history of obesity	Confounder	Baseline	Not predicted	Binary	
Control vs intervention region	Confounder	Baseline	Not predicted	Binary	
Baseline maternal BMI	Confounder	Baseline	Not predicted	Continuous	
Baseline screen time	Confounder	Baseline	Not predicted	Continuous	
Baseline membership in sports club	Confounder	Baseline	Not predicted	Binary	
Baseline Sleep duration	Confounder	Baseline	Not predicted	Continuous	
Baseline eating while doing something else	Confounder	Baseline	Not predicted	Binary	

Baseline SSB consumption frequency	Confounder	Baseline	Not predicted	Continuous	
**Time-varying factors**					
Time since baseline	Covariate	Post-baseline	Truncated regression	Continuous	lag1qdc
Maternal BMI	Confounder	Post-baseline	Tobit regression	Continuous	lag1qdc
Well-being score*	Confounder	Post-baseline	Tobit regression	Continuous	lag1qdc
Screen time	Exposure/confounder		Tobit regression	Continuous	lag1qdc
Membership in sports club	Exposure/confounder	Post-baseline	Logistic	Binary	lag1bin
Active transport	Exposure/confounder	Post-baseline	Logistic	Binary	lag1bin
Sleep duration	Exposure/confounder	Post-baseline	Truncated regression	Continuous	lag1qdc
Eating while doing something else	Exposure/confounder	Post-baseline	Logistic	Binary	lag1bin
SSB consumption frequency	Exposure/confounder	Post-baseline	Logistic and linear	Continuous	lag1bin
**Other important factors that could not be considered**		**Reason not accounting for the variable**			
Pubertal status	Confounder	Only assessed in subgroup at W2			

Table S4a: Functional form and type of model chosen for the covariates when being used as predictor/ response variable in the 6-year and 13-year analyses based on the entire study sample

#Details are given in the user guide of the GFORMULA SAS macro available at *www.hsph.harvard.edu/causal/software*

*Well-being score was only assessed from W0 to W2, but not in W3, and was hence only considered in the 6-year analyses

Variable	Purpose	Time period of variable assessment	Type of model when used as dependent variable	Functional form when used as predictor	Modelling type selected in SAS macro[#] for covariate history (covXptype)
**Baseline**					
Age at cohort entry	Confounder	Baseline	Not predicted	Continuous	
Age squared					
Age cubic					
Sex	Confounder	Baseline	Not predicted	Binary	
Region of residence	Confounder	Baseline	Not predicted	Categorical (3 categories)	
Parental educational level	Confounder	Baseline	Not predicted	Categorical (3 categories)	
Migrant status	Confounder	Baseline	Not predicted	Binary	
Pregnancy weight gain	Confounder	Baseline	Not predicted	Continuous	
Maternal age at birth	Confounder	Baseline	Not predicted	Continuous	
Indicator for preterm birth	Confounder	Baseline	Not predicted	Binary	
Smoking during pregnancy	Confounder	Baseline	Not predicted	Binary	
Total breast feeding duration	Confounder	Baseline	Not predicted	Continuous	
Baseline energy intake	Confounder	Baseline	Not predicted	Continuous	
Family history of obesity	Confounder	Baseline	Not predicted	Binary	
Control vs intervention region	Confounder	Baseline	Not predicted	Binary	
Baseline maternal BMI	Confounder	Baseline	Not predicted	Continuous	
Baseline screen time	Confounder	Baseline	Not predicted	Continuous	
Baseline Sleep duration	Confounder	Baseline	Not predicted	Continuous	
Baseline SSB consumption frequency	Confounder	Baseline	Not predicted	Continuous	
Baseline MVPA	Confounder	Baseline	Not predicted	Continuous	

Baseline LPA	Confounder	Baseline	Not predicted	Continuous	
**Time-varying factors**					
Time since baseline	Covariate	Post-baseline	Truncated regression	Continuous	lag1qdc
Maternal BMI	Confounder	Post-baseline	Tobit regression	Continuous	lag1qdc
Well-being score*	Confounder	Post-baseline	Tobit regression	Continuous	lag1qdc
Screen time	Confounder	Post-baseline	Tobit regression	Continuous	lag1qdc
Valid wear time	Confounder	Post-baseline	Tobit regression	Continuous	lag1bin
LPA	Confounder	Post-baseline	Tobit regression	Continuous	lag1bin
MVPA	Exposure	Post-baseline	Tobit regression	Continuous	lag1bin
Sleep duration	Confounder	Post-baseline	Truncated regression	Continuous	lag1qdc
SSB consumption frequency	Confounder	Post-baseline	Logistic and linear	Continuous	lag1bin
**Other important factors that could not be considered**		**Reason not accounting for the variable**			
Pubertal status	Confounder	Only assessed in subgroup at W2			

Table S4b: Functional form and type of model chosen for the covariates when being used as predictor/ response variable in the analyses for interventions on MVPA based on the subgroup with accelerometer data

#Details are given in the user guide of the GFORMULA SAS macro available at *www.hsph.harvard.edu/causal/software*

*Well-being score was only assessed in W0 to W2, but not in W3, and was hence only considered in the 6-year analyses

Supplementary Material S5

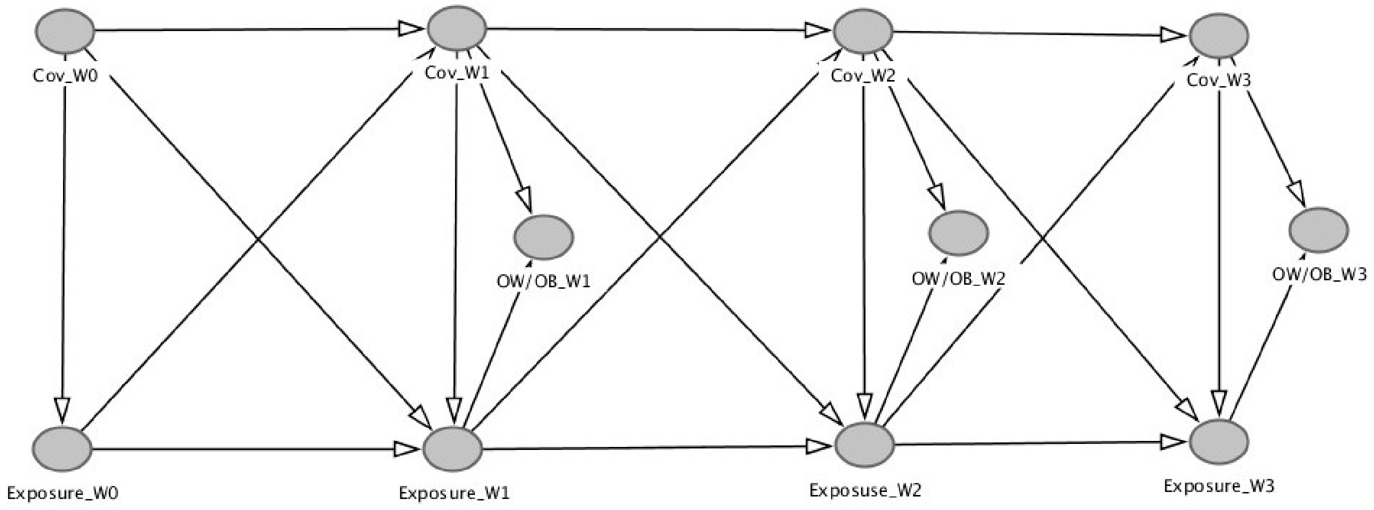

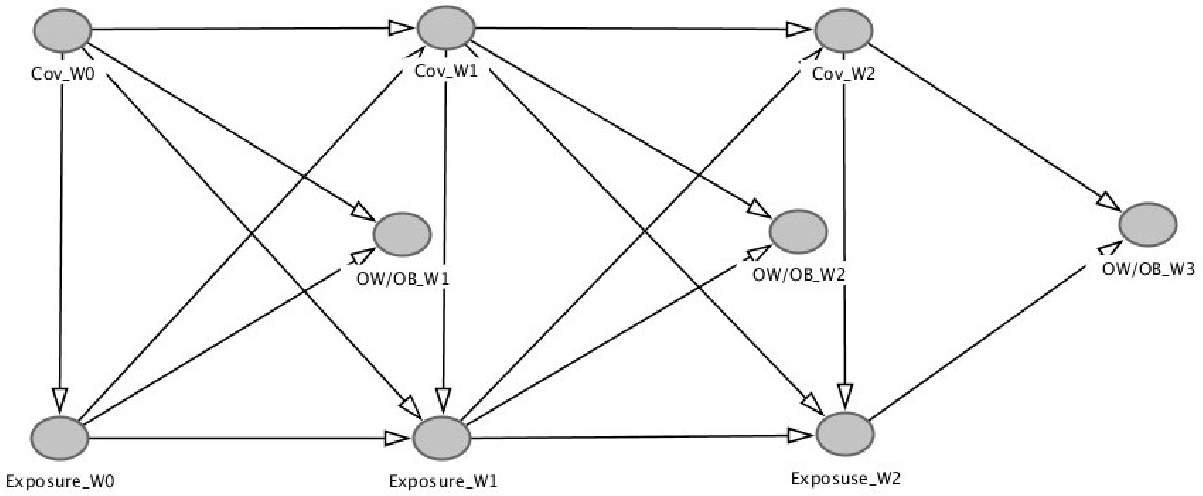

Figure S5: Assumed time-order between covariates, exposures and outcome in model allowing contemporaneous effects (upper panel) and in model allowing only time-delayed effects (lower panel); arrows e.g. from covariates at W0 to covariates/exposures/outcomes at later time points were omitted for clarity

Supplementary Material S6

	W0		W1		W2		W3	
	**N**	**%**	**N**	**%**	**N**	**%**	**N**	**%**
**Total number of study subjects**	10877		6871		3023		1466	
**Adherence to all recommendations**	810	7.5	780	11.4	179	5.9	45	3.1
**Sleep recommendation**	8972	82.5	6023	87.7	2365	78.2	1002	68.4
**Screen time recommendation**	5881	54.1	3577	52.1	988	32.7	340	23.2
**Use of active form of transport**	4258	39.2	3051	44.4	1405	46.5	1083	73.9
**SSB recommendation**	8924	82.0	5904	85.9	1873	62.0	836	57.0
**Membership in a sports club**	4727	43.5	4360	63.5	2194	72.6	912	62.2
**Non-daily eating while doing something else**	8854	81.4	5619	81.8	2130	70.5	689	47.0

Table S6a: Numbers and percentages of children adhering observationally to the different recommendations at W0 to W3 as well as numbers and percentages of children adhering to all recommendations

	W0		W1		W2	
	**N**	**%**	**N**	**%**	**N**	**%**
**Total number of study subjects**	2203		2061		1243	
**Adhere to MVPA recommendation**	678	30.78	800	38.82	453	36.44

Table S6b: Numbers and percentages of children adhering to MVPA recommendation at W0 to W2

Supplementary Material S7: Graphical display of population risk differences and 95% confidence intervals using the g-formula when intervening only on children of mother's with BMI > 25 kg/m^2 and children of parents with low/medium ISCED level at baseline

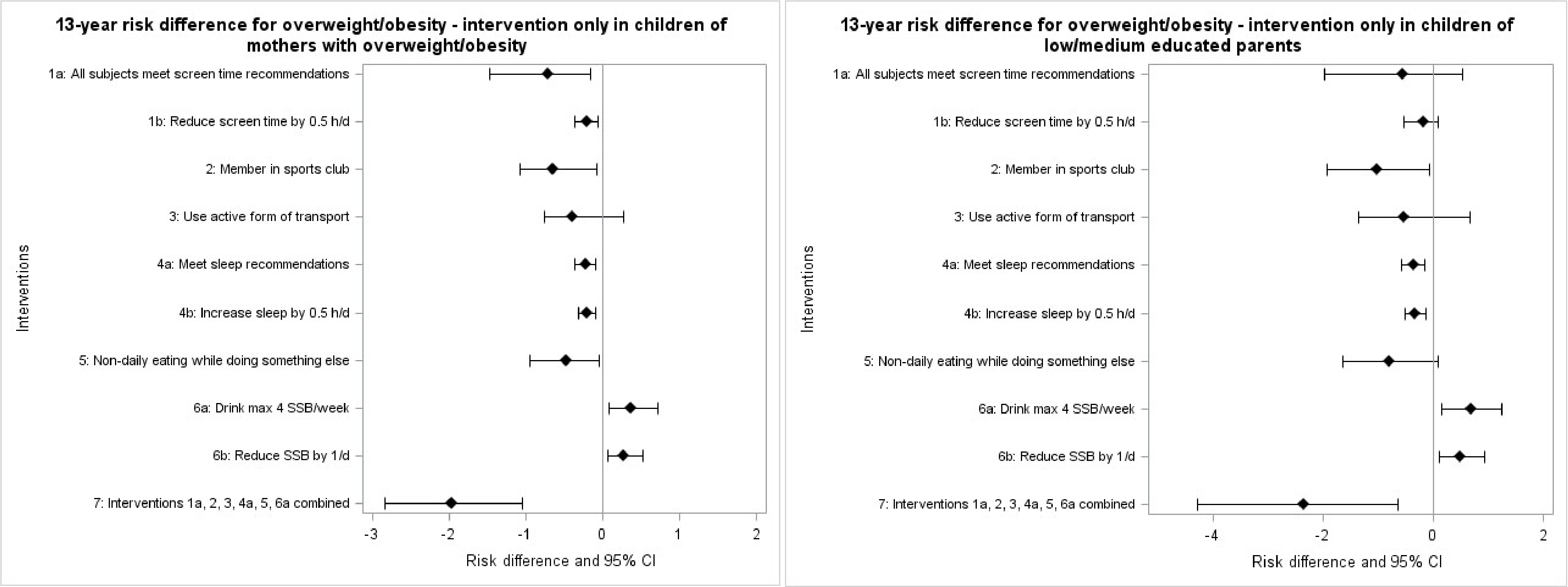

Figure S7: Population risk differences and 95% confidence intervals using the g-formula. Hypothetical interventions on children of mother's with BMI > 25 kg/m^2 (left panel) and children of parents with low/medium ISCED level at baseline (right panel) using data from W0 to W3. Model allowing contemporaneous effects of exposures on the outcome.

Supplementary Material S8: Intervention effects on risk of developing overweight/obesity when intervening only on males/females or younger/older children

	Intervention effects when intervening only on males	Risk (%)	95% LCL	95% UCL	Population risk ratio	95% LCL	95% UCL	Population risk difference	95% LCL	95% UCL	Cumulative % intervened[a]	Average % intervened[b]
0	Natural course	30.7	28.4	32.7	**1**	1	1	**0**	0	0	0.0	0.0
1a	All subjects meet screen time recommendations	**30.2**	27.6	32.0	**0.98**	0.94	1.03	**-0.51**	-1.98	0.83	50.7	40.6
1b	All subjects reduce screen time by 0.5 hours/day	**30.5**	28.3	32.7	**1**	0.98	1	**-0.15**	-0.45	0.14	50.7	40.6
2	All subjects are members in sports club	**29.2**	26.9	31.5	**0.95**	0.93	0.99	**-1.47**	-2.29	-0.42	39.3	15.9
3	All subjects use an active form of transport	**30.1**	27.9	32.1	**0.98**	0.96	1.01	**-0.61**	-1.13	0.28	40.1	18.4
4a	All subjects meet sleep recommendations	**30.4**	27.9	32.2	**0.99**	0.98	1	**-0.32**	-0.58	-0.08	30.8	19.9
4b	All subjects increase sleep by 0.5 hours	**30.4**	27.9	32.2	**0.99**	0.98	1	**-0.29**	-0.52	-0.07	30.8	19.9
5	All subjects non-daily eat while doing something else	**29.5**	27.0	31.5	**0.96**	0.93	0.99	**-1.19**	-2.26	-0.35	37.7	15.2
6a	All subjects drink max 4 SSB per week	**31.1**	28.6	33.1	**1.01**	1	1.03	**0.44**	-0.05	1.01	41.5	25.0
6b	All subjects reduce SSB by 1/day	**31.0**	28.5	33.0	**1.01**	1	1.03	**0.33**	-0.03	0.75	41.5	25.0
7	All subjects adhere to recommendation for all variables	**27.4**	24.4	30.0	**0.89**	0.85	0.95	**-3.25**	-4.58	-1.64	51.6	48.4

Table S8a: Population risk estimates using the g-formula. Hypothetical interventions on male subjects using data from W0 to W3 allowing contemporaneous effects of exposures on the outcome

[a] The cumulative percent intervened on is the percent of the population required to change behavior in at least one wave

[b]The average percent intervened on is the average, across all waves, of the percent of the study population required to change behavior in a given wave

	Intervention effects when intervening only on females	Risk (%)	95% LCL	95% UCL	Population risk ratio	95% LCL	95% UCL	Population risk difference	95% LCL	95% UCL	Cumulative % intervened[a]	Average % intervened[b]
0	Natural course	30.7	28.4	32.7	**1**	1	1	**0**	0	0	0.0	0.0
1a	All subjects meet screen time recommendations	**29.0**	26.6	30.6	**0.94**	0.91	0.98	**-1.69**	-2.67	-0.59	46.3	34.5
1b	All subjects reduce screen time by 0.5 hours/day	**30.2**	28.0	32.3	**0.98**	0.98	0.99	**-0.49**	-0.74	-0.18	46.3	34.5
2	All subjects are members in sports club	**30.4**	28.0	32.5	**0.99**	0.97	1.03	**-0.26**	-1.03	0.79	37.6	15.9
3	All subjects use an active form of transport	**30.2**	28.0	31.9	**0.98**	0.96	1.01	**-0.51**	-1.15	0.27	37.6	17.6
4a	All subjects meet sleep recommendations	**30.3**	28.0	32.4	**0.99**	0.98	1	**-0.33**	-0.56	-0.03	27.7	17.8
4b	All subjects increase sleep by 0.5 hours	**30.4**	28.1	32.4	**0.99**	0.98	1	**-0.3**	-0.5	-0.02	27.7	17.8
5	All subjects non-daily eat while doing something else	**30.7**	28.3	32.8	**1**	0.97	1.03	**0.07**	-0.83	1.03	33.1	12.8
6a	All subjects drink max 4 SSB per week	**31.2**	29.0	33.2	**1.02**	1	1.03	**0.53**	-0.05	1.01	36.6	21.5
6b	All subjects reduce SSB by 1/day	**31.1**	28.8	33.1	**1.01**	1	1.02	**0.4**	-0.03	0.78	36.6	21.5
7	All subjects adhere to recommendation for all variables	**28.6**	25.8	30.7	**0.93**	0.88	0.98	**-2.06**	-3.56	-0.56	48.4	44.7

Table S8b: Population risk estimates using the g-formula. Hypothetical interventions on female subjects using data from W0 to W3 allowing contemporaneous effects of exposures on the outcome

[a] The cumulative percent intervened on is the percent of the population required to change behavior in at least one wave

[b]The average percent intervened on is the average, across all waves, of the percent of the study population required to change behavior in a given wave

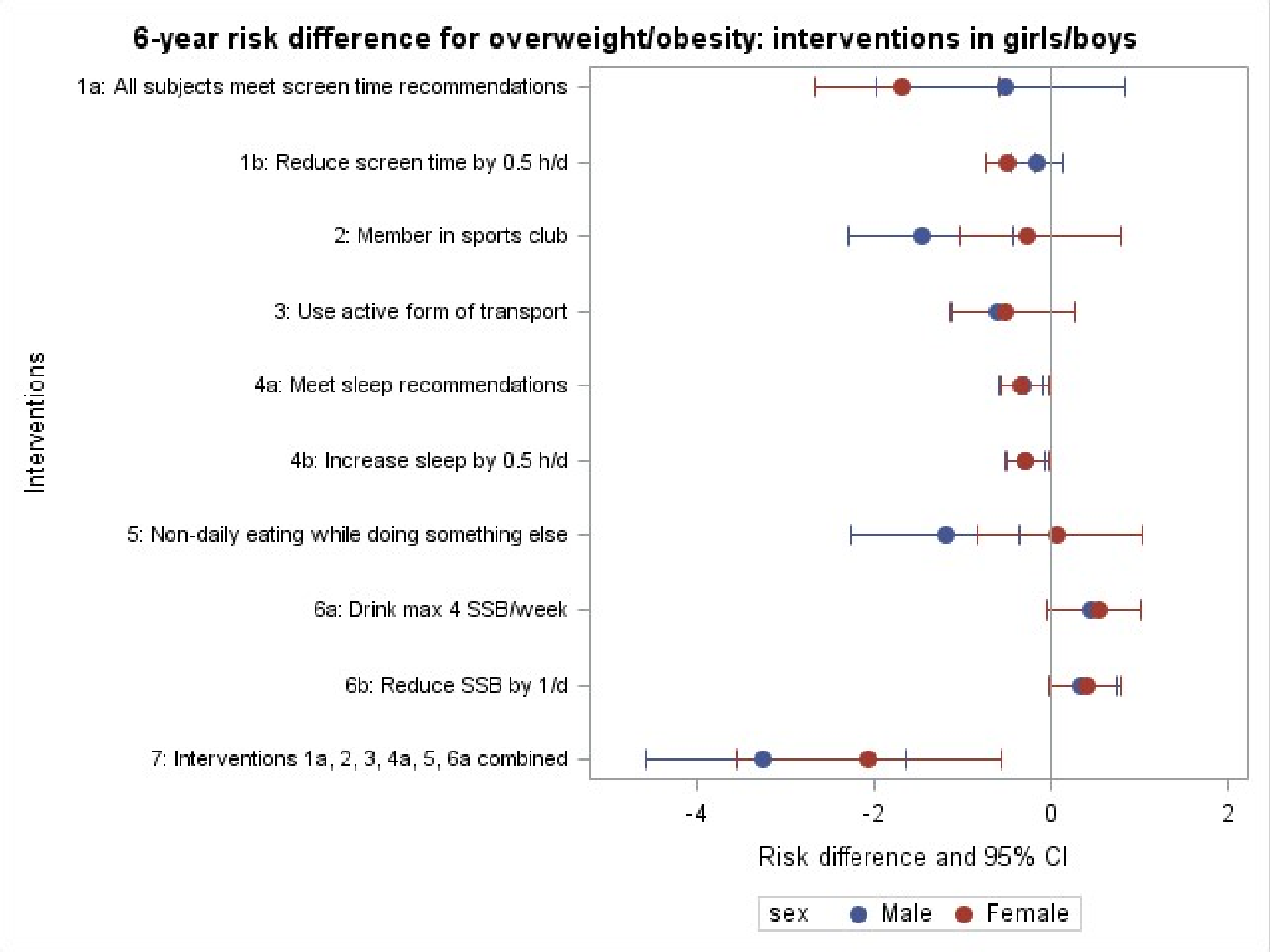

Figure S8c: Graphical display of population risk differences and 95% confidence intervals using the g-formula when intervening only in girls or when intervening only in boys

	Intervention effects when intervening only on < 6 year olds	Risk (%)	95% LCL	95% UCL	Population risk ratio	95% LCL	95% UCL	Population risk difference	95% LCL	95% UCL	Cumulative % intervened[a]	Average % intervened[b]
0	Natural course	30.7	28.4	32.7	**1**	1	1	**0**	0	0	0.0	0.0
1a	All subjects meet screen time recommendations	**28.8**	26.5	30.6	**0.94**	0.9	0.97	**-1.85**	-3.08	-0.86	48.4	39.4
1b	All subjects reduce screen time by 0.5 hours/day	**30.1**	28.0	32.2	**0.98**	0.97	0.99	**-0.52**	-0.87	-0.28	48.4	39.4
2	All subjects are members in sports club	**30.6**	28.3	32.5	**1**	0.97	1.03	**-0.1**	-1	0.84	42.4	17.9
3	All subjects use an active form of transport	**30.1**	27.6	31.8	**0.98**	0.95	1.02	**-0.57**	-1.42	0.68	40.5	19.0
4a	All subjects meet sleep recommendations	**30.3**	27.8	32.2	**0.99**	0.98	0.99	**-0.39**	-0.65	-0.17	34.2	23.8
4b	All subjects increase sleep by 0.5 hours	**30.3**	27.9	32.3	**0.99**	0.98	1	**-0.34**	-0.55	-0.14	34.2	23.8
5	All subjects non-daily eat while doing something else	**30.1**	27.9	32.4	**0.98**	0.95	1.01	**-0.58**	-1.46	0.3	34.2	13.5
6a	All subjects drink max 4 SSB per week	**31.0**	28.7	33.1	**1.01**	1	1.03	**0.32**	-0.09	0.75	36.7	21.2
6b	All subjects reduce SSB by 1/day	**30.9**	28.6	33.0	**1.01**	1	1.02	**0.25**	-0.06	0.58	36.7	21.2
7	All subjects adhere to recommendation for all variables	**27.8**	25.0	29.9	**0.91**	0.86	0.97	**-2.84**	-4.18	-0.99	50.0	47.2

Table S8d: Population risk estimates using the g-formula. Hypothetical interventions on subjects aged < 6 years at baseline using data from W0 to W3 allowing contemporaneous effects of exposures on the outcome

[a] The cumulative percent intervened on is the percent of the population required to change behavior in at least one wave

[b]The average percent intervened on is the average, across all waves, of the percent of the study population required to change behavior in a given wave

	Intervention effects when intervening only on ≥ 6 year olds	**Risk (%)**	**95% LCL**	**95% UCL**	**Population risk ratio**	**95% LCL**	**95% UCL**	**Population risk difference**	**95% LCL**	**95% UCL**	**Cumulative % intervened[a]**	**Average % intervened[b]**
0	Natural course	30.7	28.4	32.7	**1**	1	1	**0**	0	0	0.0	0.0
1a	All subjects meet screen time recommendations	**30.3**	28.0	32.5	**0.99**	0.95	1.03	**-0.36**	-1.44	0.85	48.7	35.7
1b	All subjects reduce screen time by 0.5 hours/day	**30.6**	28.2	32.7	**1**	0.99	1.01	**-0.12**	-0.37	0.15	48.7	35.7
2	All subjects are members in sports club	**29.2**	26.7	31.3	**0.95**	0.93	0.97	**-1.5**	-2.21	-0.87	34.8	14.1
3	All subjects use an active form of transport	**30.1**	27.6	32.4	**0.98**	0.95	1.01	**-0.55**	-1.51	0.38	37.9	16.9
4a	All subjects meet sleep recommendations	**30.4**	28.0	32.4	**0.99**	0.99	1	**-0.26**	-0.41	-0.11	24.3	13.9
4b	All subjects increase sleep by 0.5 hours	**30.4**	28.0	32.4	**0.99**	0.99	1	**-0.25**	-0.4	-0.11	24.3	13.9
5	All subjects non-daily eat while doing something else	**30.0**	27.6	32.4	**0.98**	0.95	1.01	**-0.7**	-1.55	0.23	36.2	14.4
6a	All subjects drink max 4 SSB per week	**31.3**	28.9	33.3	**1.02**	1	1.04	**0.65**	0.11	1.22	41.3	25.2
6b	All subjects reduce SSB by 1/day	**31.1**	28.8	33.2	**1.02**	1	1.03	**0.48**	0.09	0.93	41.3	25.2
7	All subjects adhere to recommendation for all variables	**28.2**	25.6	31.1	**0.92**	0.86	0.98	**-2.48**	-4.17	-0.57	50.0	45.9

Table S8e: Population risk estimates using the g-formula. Hypothetical interventions on subjects aged ≥ 6 years at baseline using data from W0 to W3 allowing contemporaneous effects of exposures on the outcome

[a] The cumulative percent intervened on is the percent of the population required to change behavior in at least one wave

[b]The average percent intervened on is the average, across all waves, of the percent of the study population required to change behavior in a given wave

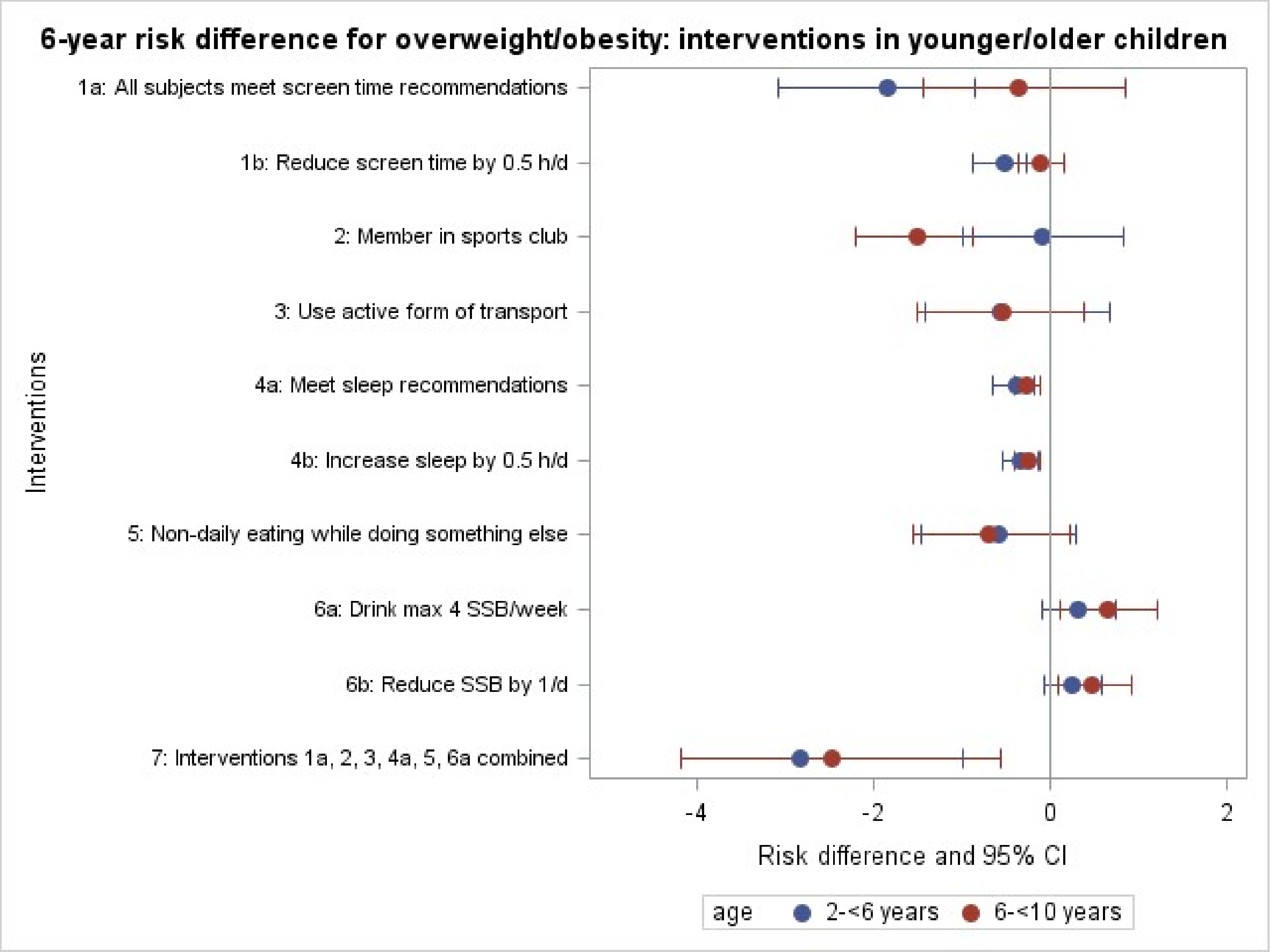

Figure S8f: Graphical display of population risk differences and 95% confidence intervals using the g-formula when intervening only in children 2 to <6 years vs in children 6 to <10 years

Supplementary Material S9

**Only time-delayed effects**<br>Observed risk: 31.4	**Risk (%)**	**95% LCL**	**95% UCL**	**Population risk ratio**	**95% LCL**	**95% UCL**	**Population risk difference**	**95% LCL**	**95% UCL**	**Cumulative % intervened[a]**	**Avererage % intervened[b]**
0 Natural course	**31.0**	29.7	32.8	**1**	1	1	**0**	0	0	0.0	0.0
1a All subjects meet screen time recommendations	**28.0**	26.1	30.1	**0.9**	0.85	0.95	**-2.98**	-4.72	-1.58	87.3	59.9
1b All subjects reduce screen time by 0.5 hours/day	**30.0**	28.7	31.7	**0.97**	0.95	0.98	**-0.96**	-1.45	-0.51	87.3	59.9
2 All subjects are members in sports club	**29.8**	27.5	32.7	**0.96**	0.91	1.02	**-1.19**	-2.62	0.62	66.4	30.9
3 All subjects use an active form of transport	**30.4**	28.0	32.9	**0.98**	0.92	1.04	**-0.62**	-2.56	1.05	77.8	37.4
4a All subjects meet sleep recommendations	**30.9**	29.7	32.7	**1**	0.99	1	**-0.14**	-0.26	-0.01	15.6	13.6
4b All subjects increase sleep by 0.5 hours	**30.9**	29.7	32.7	**1**	0.99	1	**-0.13**	-0.26	-0.01	15.6	13.6
5 All subjects non-daily eat while doing something else	**30.5**	28.4	32.6	**0.98**	0.95	1.03	**-0.51**	-1.75	0.8	43.9	20.2
6a All subjects drink max 4 SSB per week	**31.0**	29.5	32.9	**1**	0.98	1.02	**-0.01**	-0.6	0.51	58.0	33.6
6b All subjects reduce SSB by 1/day	**31.0**	29.6	32.9	**1**	0.99	1.01	**0**	-0.45	0.39	58.0	33.6
7 All subjects adhere to recommendation for all variables	**26.6**	23.4	29.9	**0.86**	0.77	0.96	**-4.41**	-7.03	-1.35	98.5	78.4

Table S9a: Population risk estimates using the g-formula. Hypothetical interventions on entire cohort using data from W0 to W3 allowing only time-delayed (but not contemporaneous) effects of exposures on the outcome

[a] The cumulative percent intervened on is the percent of the population required to change behavior in at least one wave

[b]The average percent intervened on is the average, across all waves, of the percent of the study population required to change behavior in a given wave

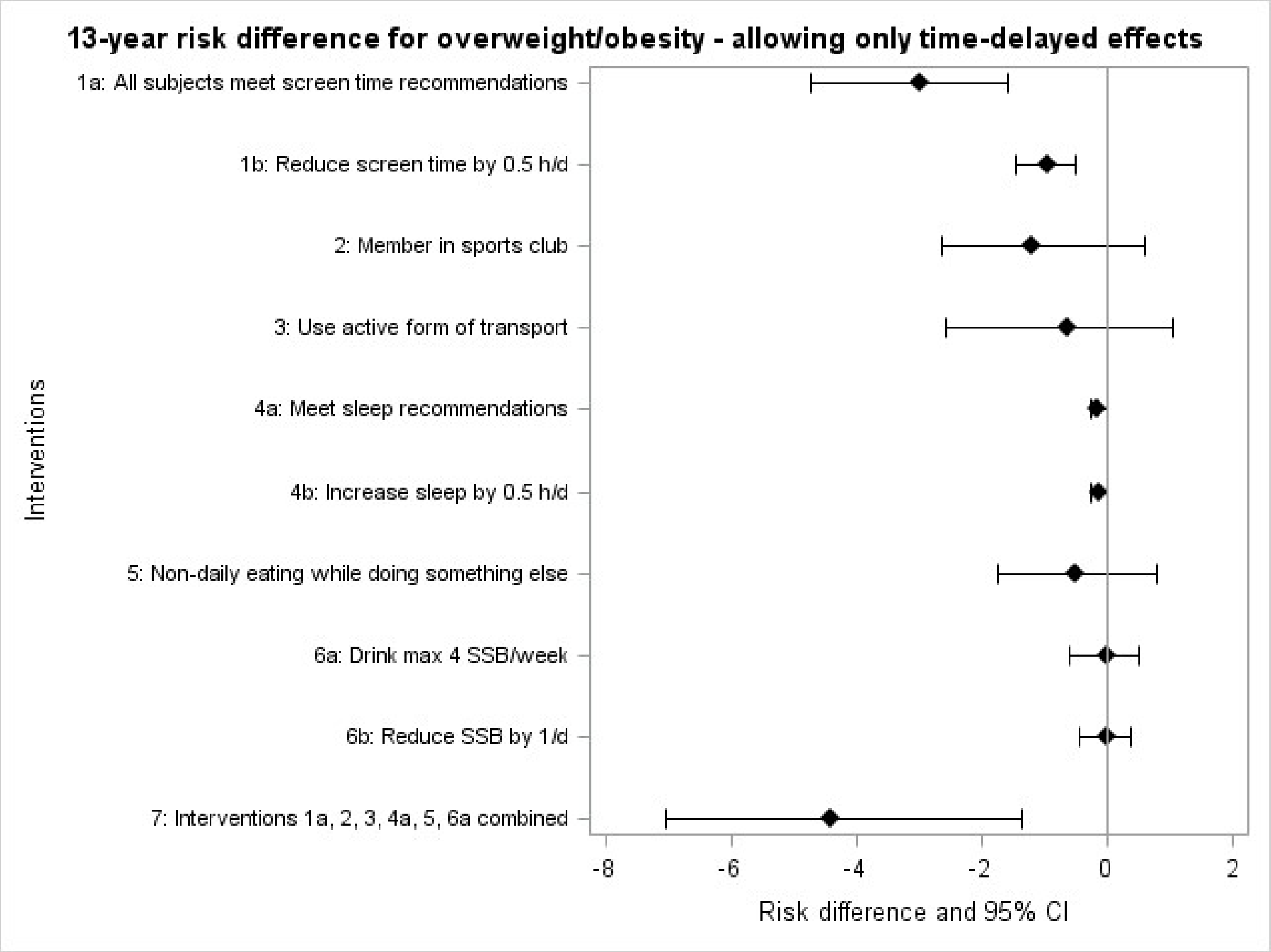

Figure S9b: Graphical display of population risk differences and 95% confidence intervals using the g-formula when allowing only time-delayed effects

Supplementary Material S10

	Effects after 6 years<br>Observed risk: 22.5	Risk (%)	95% LCL	95% UCL	Population risk ratio	95% LCL	95% UCL	Population risk difference	95% LCL	95% UCL	Cumulative % intervened on[a]	Average % intervened on[b]
0	Natural course	**22.5**	21.3	23.7	**1**	1	1	**0**	0	0	0.0	0.0
1a	All subjects meet screen time recommendations	**20.1**	18.2	21.6	**0.90**	0.85	0.94	**-2.33**	-3.61	-1.33	89.4	68.3
1b	All subjects reduce screen time by 0.5 hours/day	**21.6**	20.2	22.8	**0.96**	0.94	0.98	**-0.85**	-1.26	-0.44	89.4	68.3
2	All subjects are members in sports club	**21.3**	19.5	22.8	**0.95**	0.91	0.99	**-1.17**	-2.09	-0.26	70.0	33.7
3	All subjects use an active form of transport	**21.6**	19.8	23.6	**0.96**	0.92	1.03	**-0.84**	-1.82	0.61	78.8	37.8
4a	All subjects meet sleep recommendations	**22.0**	20.7	23.3	**0.98**	0.96	0.99	**-0.49**	-0.83	-0.25	47.1	32.2
4b	All subjects increase sleep by 0.5 hours	**22.0**	20.8	23.3	**0.98**	0.97	0.99	**-0.45**	-0.73	-0.22	47.1	32.2
5	All subjects non-daily eat while doing something else	**22.2**	20.9	23.8	**0.99**	0.95	1.02	**-0.31**	-1.09	0.5	46.8	21.1
6a	All subjects drink max 4 SSB per week	**23.2**	21.2	25.3	**1.03**	0.99	1.09	**0.76**	-0.12	1.83	90.9	75.4
6b	All subjects reduce SSB by 1/day	**23.0**	21.3	24.7	**1.02**	1	1.06	**0.51**	-0.09	1.22	90.9	75.4
7	All subjects adhere to recommendation for all variables	**18.6**	16.4	20.5	**0.83**	0.74	0.9	**-3.89**	-5.95	-2.28	99.9	94.2

Table S10a: Population risk estimates using the g-formula. Hypothetical interventions on entire cohort using data from W0 to W2, i.e. over 6-year period, allowing contemporaneous effects of exposures on the outcome

[a] The cumulative percent intervened on is the percent of the population required to change behavior in at least one wave

[b]The average percent intervened on is the average, across all waves, of the percent of the study population required to change behavior in a given wave

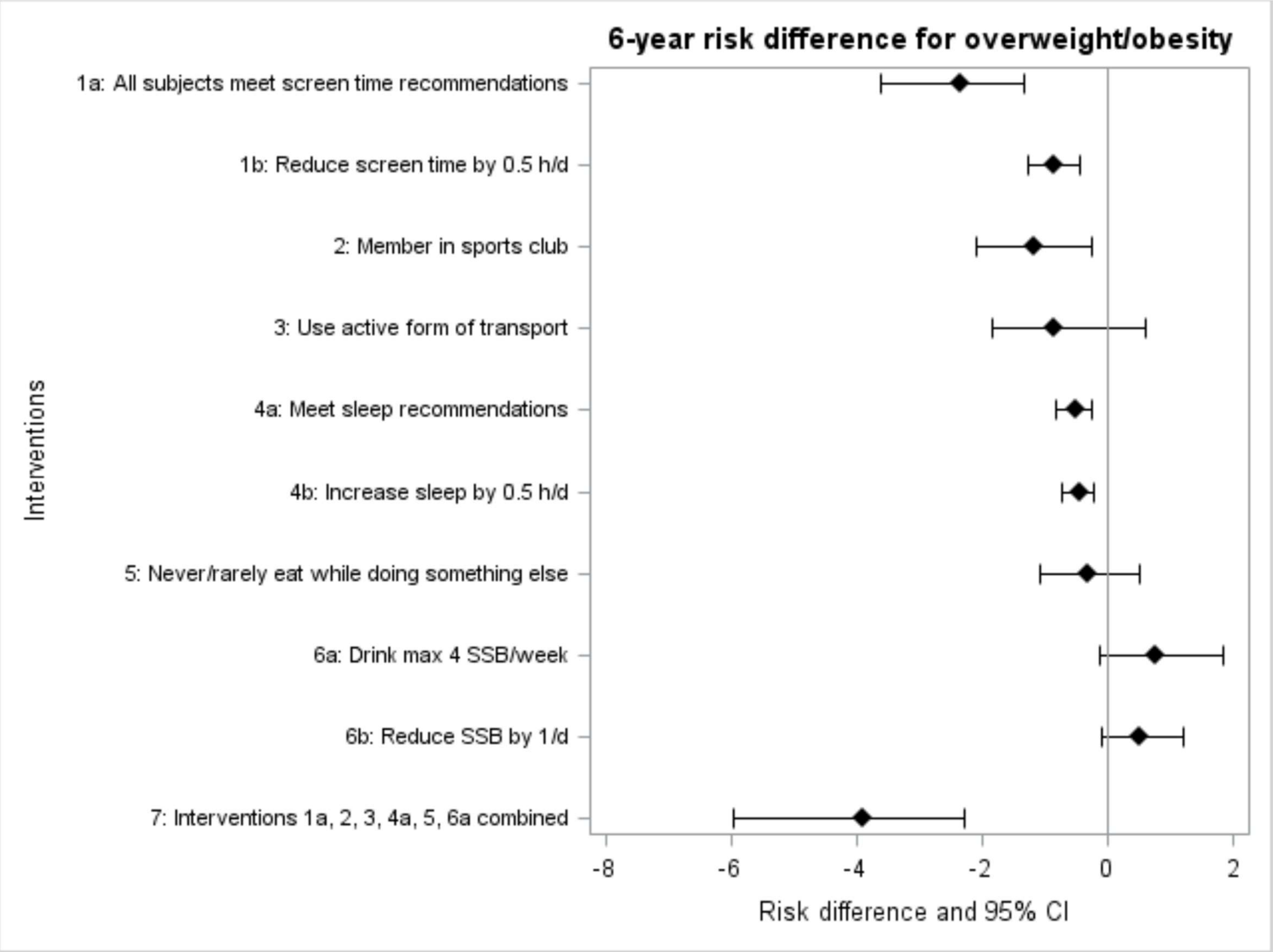

Figure S10b: Graphical display of population risk differences and 95% confidence intervals using the g-formula: estimates over a 6-year period

Supplementary Material S11: Sensitivity analysis adding the distance to school/kindergarten/work as an additional time-varying covariate

The distance to school/kindergarten is certainly a predictor of using an active form of transport, i.e. related to our exposure of interest. However, another necessary condition for a confounder is that the confounder (distance) is separately predictive (given the other confounders already included) of the outcome of interest (OW/OB in our case). As we do not believe that the latter condition is met, we opted to not adjust for the distance to school/kindergarten in our main analyses, but did so in a sensitivity analysis:

In a first step, we harmonized our measures of distance to school/kindergarten at the different waves. In W0/W1, the distance was queried based on a multiple-choice question. Continuous values were assigned to the categories as follows:
‘0 to 1 km’= 0.5,
‘1 to 2 km’= 1.5,
‘2 to 3 km’= 2.5,
‘3 to 4 km’= 3.5,
‘More than 4 km’= 4.5
In W2/W3 the distance was queried based on an open question (continuous in km). The values were truncated at 10 km as the categories at W0/W1 did not capture high distances and the skewness led to convergence problems when applying the parametric g-formula. We then added this harmonized variable (distance to work/school/kindergarten in km) as a time-varying covariate to our main model.

The effect of active transport on the incidence of OW/OB increased (risk difference: 1.99 [-3.80;-0.29]) while effects of all single interventions remained similar. The effect of the joint intervention increased (corresponding to the increase of the effect of active transport; risk difference 6.02 [-9.01;-3.82]).

References

1. Cole TJ, Lobstein T. Extended international (IOTF) body mass index cut-offs for thinness, overweight and obesity. Pediatr Obes. 2012;7(4):284-94.
2. World Health Organization. Obesity and overweight. In: Fact Sheets. World Health Organization. 2021. https://www.who.int/news-room/fact-sheets/detail/obesity-and-overweight. Accessed 16 August 2022.
3. United Nations Educational Scientific and Cultural Organization (UNESCO). International Standard Classification of Education, ISCED 2011. Canada: UNESCO Institute for Statistics2012.
4. Bullinger M, Brutt AL, Erhart M, Ravens-Sieberer U, Group BS. Psychometric properties of the KINDL-R questionnaire: results of the BELLA study. Eur Child Adolesc Psychiatry. 2008;17 Suppl 1:125-32.
5. Ravens-Sieberer U, Bullinger M. Kindl-R English questionnaire for measuring health-related quality of life in children and adolescents. Revised Version Manual. . Ulrike Ravens-Sieberer & Monika Bullinger; 2000.
6. Hense S, Barba G, Pohlabeln H, et al. Factors that influence weekday sleep duration in European children. Sleep. 2011;34(5):633-9.
7. Konstabel K, Veidebaum T, Verbestel V, et al. Objectively measured physical activity in European children: the IDEFICS study. Int J Obes (Lond). 2014;38 Suppl 2:S135-43.
8. Choi L, Liu Z, Matthews CE, Buchowski MS. Validation of accelerometer wear and nonwear time classification algorithm. Med Sci Sports Exerc. 2011;43(2):357-64.
9. Wirsik N, Otto-Sobotka F, Pigeot I. Modeling physical activity data using L(0) -penalized expectile regression. Biom J. 2019;61(6):1371-84.
10. Evenson KR, Catellier DJ, Gill K, Ondrak KS, McMurray RG. Calibration of two objective measures of physical activity for children. J Sports Sci. 2008;26(14):1557-65.
11. Young JG, Cain LE, Robins JM, O'Reilly EJ, Hernan MA. Comparative effectiveness of dynamic treatment regimes: an application of the parametric g-formula. Stat Biosci. 2011;3(1):119-43.
